# Simple, Fast, and Ultrasensitive Fluorescent Detection of Peroxymonosulfate in Chemical, Enzymatic, and Cellular Systems

**DOI:** 10.1021/acs.analchem.6c03097

**Published:** 2026-09-22

**Authors:** Radosław Podsiadły, Aleksandra Grzelakowska, Kumari Neelam, Radosław Michalski, Michał Rostkowski, Renata Smulik-Izydorczyk, Adam Sikora, Jacek Zielonka

**Affiliations:** † Institute of Polymer and Dye Technology, Faculty of Chemistry, Lodz University of Technology, Stefanowskiego 16, 90-537 Lodz, Poland; ‡ Institute of Applied Radiation Chemistry, Faculty of Chemistry, Lodz University of Technology, Zeromskiego 116, 90-924 Lodz, Poland; § Department of Biophysics and Redox & Bioenergetics Core, Cancer Center Translational Metabolomics Shared Resource, 5506 Medical College of Wisconsin, 8701 Watertown Plank Road, Milwaukee, Wisconsin 53226, United States

## Abstract

Peroxymonosulfate (PMS) is widely used in advanced oxidation processes (AOP) to remove organic contaminants from wastewater. A simple method for determining PMS is necessary to dose this oxidant effectively and use it adequately in AOP. Here, we report a simple, fast, and accurate method for quantifying PMS using a pro-fluorescent boronate redox probe. In contrast to previously described methods, the proposed assay does not require activation of PMS by a low-valent transition metal. Based on the determined second-order rate constant of the direct oxidation reaction of the coumarin-7-boronic acid probe with PMS (^2^ *k* = 1.1 × 10^4^ M^–1^ s^–1^), we have developed a method based on high-performance liquid chromatography coupled with fluorescence detection (HPLC-FLD) for ultrasensitive detection of PMS with a detection limit of 5 nM and a quantification limit of 40 nM. The HPLC-FLD method enables detection of PMS in real water samples, confirming its practical usefulness. The reported method enables the determination of PMS over a wide pH range and in the presence of common ions. It also enables tracking of PMS formation in sulfite solutions. Using activated RAW 264.7 macrophages, we demonstrate the feasibility of real-time monitoring of PMS production using a plate reader with fluorescence detection, along with appropriate control experiments.

## Graphical abstract

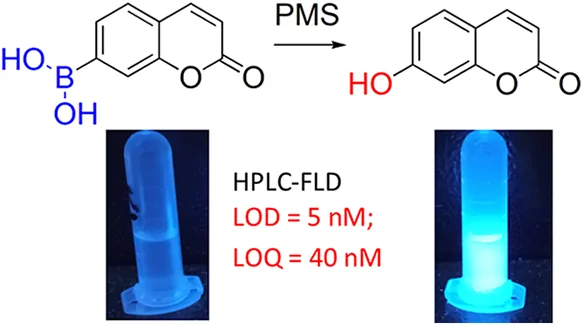

## Introduction

Peroxymonosulfate (PMS, HSO_5_^–^) is a strong oxidant with a standard two-electron reduction potential of 1.81 V.^1^ It is used in advanced oxidation processes (AOP) due to its stability and effectiveness in removing contaminants.^2–5^ Activations of PMS by metals and chemical species are required for effective degradation of organic and inorganic contaminants, which have also been widely studied.^6,7^ PMS was also postulated as one of the reactive products formed during one-electron oxidation of (bi)­sulfite, initiated by transition metals, metalloenzymes (e.g., cytochrome *c* oxidase), O_2_, superoxide, photoexcited chromophores, and UV radiation.^8–18^ Generated in the first step of sulfite oxidation (Scheme 1), the mild oxidant sulfur trioxide radical anion (SO_3_^•–^, reaction 1) reacts with oxygen (reaction 2), forming peroxosulfate radical anion (O_3_SOO^•–^).^10,18^ This radical intermediate can react with (bi)­sulfite either via electron transfer to yield HSO_5_^–^ (PMS) and regenerate SO_3_^•–^ (reaction 3), or via oxygen atom transfer to generate another strong oxidant, SO_4_^•–^ (reaction 4). SO_4_^•–^, in turn, will react with (bi)­sulfite to produce more SO_3_^•–^ (reaction 5), further propagating (bi)­sulfite oxidation, at the expense of molecular oxygen. Due to the limited number of chemical probes to detect various intermediates formed during sulfite oxidation, researchers’ attention focused on detecting radical species using spin traps and recording EPR spectra,^8,19–24^ with only few attempts to detect and confirm PMS formation in the reaction mechanism, as detailed below. Using several methodologies, the rate constant of the reaction between SO_3_^•–^ and molecular oxygen was estimated (^2^ *k* = 4.3 × 10^7^ – 2.3 × 10^9^ M^–1^ s^–1^).^25^ Similarly, the rate constants for PMS formation in the reaction of O_3_SOO^•–^ with HSO_3_^–^ and with SO_3_^2–^ were determined to be 3.0 × 10^7^ and 1.0 × 10^8^ M^–1^ s^–1^, respectively.^26^ The formation of PMS during autoxidation of sulfite was confirmed indirectly by epoxide production from ketones^27^ and amines oxidation to nitroso compounds.^28^ More recently, several methods^29–35^ with varying levels of selectivity for determining PMS concentration were developed. However, as these assays require activation of PMS by a metal species^31^ or the use of additional chemicals, such as iodides^33^ or dyes,^32,34,35^ they may be prone to interference by various water contaminants, are not suitable for use in enzymatic or *in vivo* systems, and are limited to end-point measurements. So far, the most sensitive method for detecting PMS (LOD = 10 nM, LOQ = 33 nM) utilizes the formation of fluorescent salicylic acid (SA) in the reaction between benzoic acid (BzOH) and hydroxyl radical generated from PMS by the action of cobalt­(II) salts.^31^ However, optimal analysis conditions ([BzOH] = 20 mM, [Co^2+^] = 50 μM, pH = 5.5) prevent its use in cellular systems, with cell components expected to outcompete BzOH for the hydroxyl radical.^36^ Moreover, cobalt salts are cytotoxic: at lower concentrations, they induce apoptosis; at higher concentrations, necrosis.^37^ Thus, the usage of this method in biologically relevant systems is not practical.

**Scheme 1:**
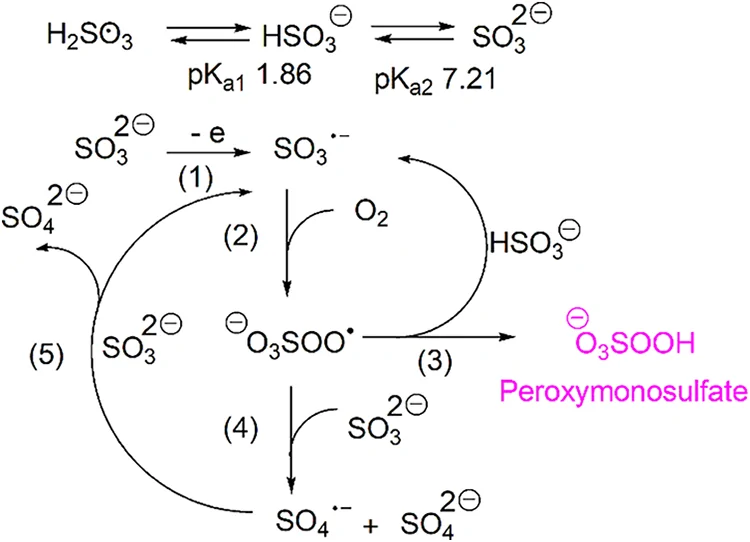
Postulated Mechanisms and Intermediates Formed during the Autoxidation of Sulfite in Aqueous Solution under Aerobic Conditions

Here, we propose a PMS detection method based on the oxidation reaction of a pro-coumarin chemosensor suitable for simple, fast, and real-time accurate analysis of peroxymonosulfate. The proposed tests also enable quick, straightforward control of the PMS content in commercially available triple salts (Oxone, 2KHSO_5_·KHSO_4_·K_2_SO_4_) and the detection of PMS formed *in situ* in cell-free and cellular systems.

## Experimental Section

### Chemicals and Reagents

PMS (Oxone, KHSO_5_·0.5 KHSO_4_·0.5K_2_SO_4_) was purchased from Fisher. Coumarin boronic acid (CBA) was synthesized using the procedure described elsewhere.^38^ Diethylenetriaminepentaacetic acid (dtpa), 7-hydroxycoumarin (COH), and other chemicals and reagents were of analytical grade and were purchased from Sigma-Aldrich. Potassium peroxydisulfate (PDS, also known as potassium persulfate) and hydrogen peroxide were purchased from Chempur (Poland).

### Experimental Procedures

The stock solutions of PMS and H_2_O_2_ were prepared each day freshly, and the concentration was determined by spectrophotometry using the extinction coefficient values of 19.1 M^–1^ cm^–1^ (at 248 nm, in water^39^) and 39.4 M^–1^ cm^–1^ (at 240 nm, in water), respectively. The oxidation reactions were carried out in a 2 mL plastic tube at room temperature (23 ± 2 °C). The appropriate probe stock solution (10 mM) in acetonitrile (typically 10 μL) was placed in a tube, and the proper volume of a phosphate buffer (0.1 M, pH 6.8) with dtpa (10 μM) was added, followed by the addition of an appropriate freshly prepared stock solution (10 mM) of PMS (typically 2–10 μL). The total volume of the reaction mixture was 1.0 mL. After a specific incubation time, the reaction mixture was diluted 10-fold into a quartz cuvette containing 0.1 M phosphate buffer (pH 6.8), and fluorescence was measured immediately. Emission spectra were collected using excitation at 325 nm, with a slit width of 0.5 nm for both excitation and emission. HPLC analyses of the reaction mixtures were conducted without dilution. The limit of detection (LOD) and limit of quantification (LOQ) were estimated based on the signal-to-noise ratio (S/N = 3 for LOD, and S/N = 10 for LOQ). All experiments were independently triplicated, and the error bars represent determined standard deviations.

### Analytical Methods

Fluorescence spectra were recorded on a spectrofluorometer FLS 920 (Edinburgh Instruments, U.K.) using aqueous solutions containing phosphate buffer (0.1 M, pH 6.8), dtpa (10 μM), and MeCN (0.1%). Absorption spectra were registered with the UV–vis-NIR spectrophotometer (Jasco V-670, Japan). The pH of the solution was measured with a pH meter (Elmetron, Poland). The products formed during PMS-induced oxidation of CBA were analyzed using a Shimadzu UHPLC instrument (Kyoto, Japan) equipped with UV–vis absorption and fluorescence detectors. The reaction mixture (10 μL) was separated using a Kinetex Biphenyl column (Phenomenex, 50 mm × 4.6 mm, 2.6 μm) that was equilibrated in a 0.1% (v/v) trifluoroacetic acid (TFA) aqueous solution containing 25% (v/v) MeCN. Gradient elution was performed at a flow rate of 1.5 mL/min with ultraviolet–visible absorption detection (λ = 330 nm) or fluorescence detection (λ_exc_ = 330 nm; λ_em_ = 450 nm). The compounds were eluted by increasing the MeCN concentration (v/v) from 25% to 100% over 6 min, followed by a further increase to 100% from 6 to 7 min. Under these conditions, CBA shows a retention time of 0.74 min and COH of 0.93 min.

### Kinetic Measurements

The stopped-flow kinetics experiments were performed using the Applied Photophysics SX-20 stopped-flow spectrometer equipped with a fluorescence detector and a thermostatically controlled cell (25 °C) with a 10 mm optical pathway. PMS (10 μM in H_2_O) was rapidly mixed (1:1 ratio) with the solution containing the CBA probe (20–100 μM), phosphate buffer (100 mM, pH 7.4), dtpa (200 μM), and MeCN (10%). The reaction mixtures were excited at 332 nm, and the intensity of the emitted light was measured at 470 nm (PMT voltage = 680 V, emission/excitation slits = 2.5 mm). The recorded kinetic traces of 7-hydroxycoumarin fluorescence signal build-up were fitted to a function corresponding to the first-order build-up kinetics (*I*(t) = *I*_max_ × (1 – e^–kt^)). Results from fitting at least three kinetic traces were averaged to obtain the pseudo-first-order rate constant. From the slope of the plot of the observed pseudo-first-order rate constant values versus the concentrations of the reactant used in excess (CBA), the second-order rate constant was derived.^40^

### Cell Culture

RAW macrophage cells (264.7 cell line) obtained from the American Type Culture Collection (Manassas, VA) were grown in high-glucose (25 mM) DMEM (Dulbecco’s Modified Eagle’s Medium) supplemented with 10% (v/v) FBS (fetal bovine serum), penicillin (100 U/mL), and streptomycin (100 μg/mL) at 37 °C in 5% carbon dioxide. The effect of the probes on cell viability was assessed using the CellTiter-Glo Luminescent Cell Viability Assay (Promega Corporation, Madison, US) based on quantitation of the total adenosine triphosphate amount, as an indicator of metabolically active cells, according to manufacturer’s instructions. Oxidation of the probes was monitored in real time in 96-well plates using fluorescence plate reader. To stimulate reduced nicotinamide adenine dinucleotide phosphate (NADPH) oxidase-dependent superoxide production, the cells were washed and then treated with phorbol 12-myristate 13-acetate (PMA, 1 μM) in DPBS (Dulbecco’s Phosphate Buffered Saline) supplemented with glucose (DPBS-G). At the time of PMA addition, the CBA probe (20 μM) and NaHSO_3_ (10 or 100 μM) were also added. Where indicated, catalase (CAT, 1 kU/mL), diphenyleneiodonium (DPI, 1 μM), or superoxide dismutase (SOD, 0.1 mg/mL) was added immediately before stimulation with PMA. Total fluorescence intensities were acquired using a CLARIOstarPlus plate reader (BMG LABTECH, Germany) with the appropriate optical settings (Ex.: 350–20 nm, auto dichroic filter, Em.: 460–20 nm). The instrument was kept at 37 °C during the measurements, and fluorescence intensity was read from the bottom of each well. Changes in the fluorescence intensity were monitored for 2 h in the kinetic mode.

### UPLC Detection of CBA and Its Oxidation Products in Culture Media

RAW 264.7 macrophages were cultured and incubated in the presence or absence of PMA, SOD, catalase, and bisulfite in DPBS in a 96-well plate, as described above. The CBA probe was present in all incubations. After a 2 h incubation, 100 μL of culture medium was transferred into Eppendorf tubes containing 100 μL of ice-cold acetonitrile with 0.1% (v/v) TFA, vortexed for 10 s, and centrifuged for 10 min at 20,000*g* and 4 °C. Next, 100 μL of the supernatant was mixed (1:1, v/v) with ice-cold 0.1% (v/v) TFA in water and centrifuged again (10 min at 20,000*g*, 4 °C). For UPLC analysis, 150 μL of the supernatant was transferred to a conical vial and analyzed by UPLC. The UPLC measurements were performed using an Acquity UPLC system (Waters Ltd.) equipped with photodiode array (PDA) and fluorescence detectors. The separation of CBA and its oxidation products was carried out on an Acquity UPLC BEH C 18 (1.7 μm, 50 mm × 2.1 mm) column equilibrated with a water/acetonitrile (90:10, v/v) mobile phase containing 0.1% (v/v) TFA. At the beginning of the analysis, the concentration of the organic phase was kept constant for 0.5 min and then increased linearly from 10 to 40% (v/v) over 2 min. An injection volume of 3.5 μL and a flow rate of 0.3 mL/min were used. The samples were maintained at 4 °C during the measurements.

### Real Water Sample

Water samples from dyeworks wastewater, Bilinski Factory of Color (Poland), Jan Ponds (51°42′38″ N, 19°28′59″E), and Grabia River (51°36′6.7″N, 19°11′11.7″E) were collected and tested in June 2026. The tap water samples were taken from the drinking water supply system on the Lodz University of Technology campus. Before analysis, samples were filtered once through 0.22 μm hydrophilic PTFE filters (LLG Spheros) and centrifuged (7000 rpm) within 10 min to remove any potential particles. Next, the known concentration of PMS was added to the water sample, and triplicate measurements were taken for each sample.

## Results and Discussion

### Oxidation of the Boronate Probe by PMS

Arylboronic acids are sensitive to nucleophilic oxidants.^41^ By analyzing the oxidation potentials of hydrogen peroxide [E°′(H_2_O_2_/H_2_O) = 1.32 V at pH 7], hypochlorous acid [E◦′(HOCl, H_2_O/Cl^–^) = 1.28 V],^42^ peroxynitrite [E°′(ONOO^–^, 2H^+^/^•^NO_2_) = 1.4 V and E°′(ONOO^–^, 2H^+^/NO_2_^–^) = 1.2 V at pH 7][Bibr ref43] and PMS [E°’(HOOSO_3_^–^/HSO_4_^–^) = 1.81 V]^1^, we reasoned that arylboronic acids should be oxidizable by PMS to form the corresponding phenols. Moreover, available kinetic data for oxidation reactions involving PMS indicate that such reactions proceed 3–4 orders of magnitude faster than those induced by hydrogen peroxide for the same substrates.^44–47^ Thus, we hypothesized that PMS would be an efficient oxidant of aromatic boronates, opening an opportunity to use boronate-based fluorogenic probes for detection and determination of PMS. To test our hypothesis, we recorded the fluorescent spectra before and after adding different amounts of PMS to the CBA probe solution in phosphate buffer. Since the stability of PMS decreases above pH 7, we first determined the efficacy of 7-hydroxycoumarin (COH) formation across different pH values. The results presented in Figure S1 indicate that the optimal pH range is between 3.6 and 10.6. Thus, we conducted the studies at a solution pH of 6.8. The fluorescence spectra observed upon oxidation of CBA by PMS (Figure 1a) were consistent with the formation of COH as the primary product (Inset in Figure 1a).

**Figure 1:**
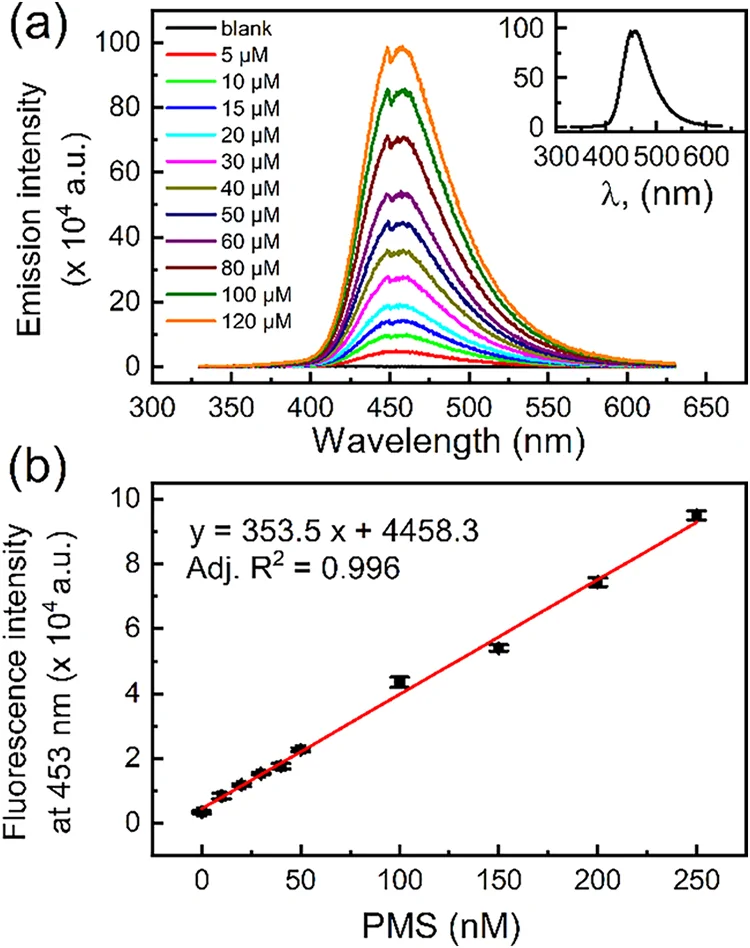
Oxidative conversion of CBA to COH induced by PMS. (a) Fluorescence spectral changes during the reaction between CBA (100 μM) and PMS (5–120 μM). Spectra were recorded after a 10-fold dilution of the reaction mixture. Inset: Fluorescent spectrum of COH (10 μM). (b) Dependence of emission intensity on the concentration of added peroxymonosulfate. Error bars indicate standard deviations of triplicates.

Next, we conducted the HPLC analyses to determine the reaction stoichiometry between CBA and PMS. The HPLC chromatograms in Figure 2a show that 7-hydroxycoumarin is the sole product formed during the oxidation of CBA by PMS. By analyzing the amounts of CBA disappearing and COH formed, it can be concluded that PMS oxidizes CBA completely when used in slight excess (Figure 2b). Less than 1.1 equiv of PMS yield 98% of COH, indicating that the PMS-induced oxidation of CBA proceeds with a 1:1 stoichiometry. It is consistent with the 1:1 stoichiometry previously reported for the oxidation of monoboronated probes by other nucleophilic oxidants, such as peroxynitrite, hypochlorous acid, amino acid hydroperoxide, and hydrogen peroxide.^41^ We assume that the oxidation of CBA proceeds with a mechanism typical for arylboronic acid and other nucleophilic oxidants, via nucleophilic addition of the negatively charged oxidant to the boron atom in ArB­(OH)_2_ with the formation of an anionic adduct. Then, the heterolytic cleavage of the O–O bond results in the elimination of an anionic good leaving group, SO_4_^2–^ (see Schemes 2 and S1).^41^ The direct and stoichiometric reaction of coumarin boronic acid with PMS provides a new, simple, and rapid analytical method for determining the amount of PMS in commercial Oxone.

**Figure 2:**
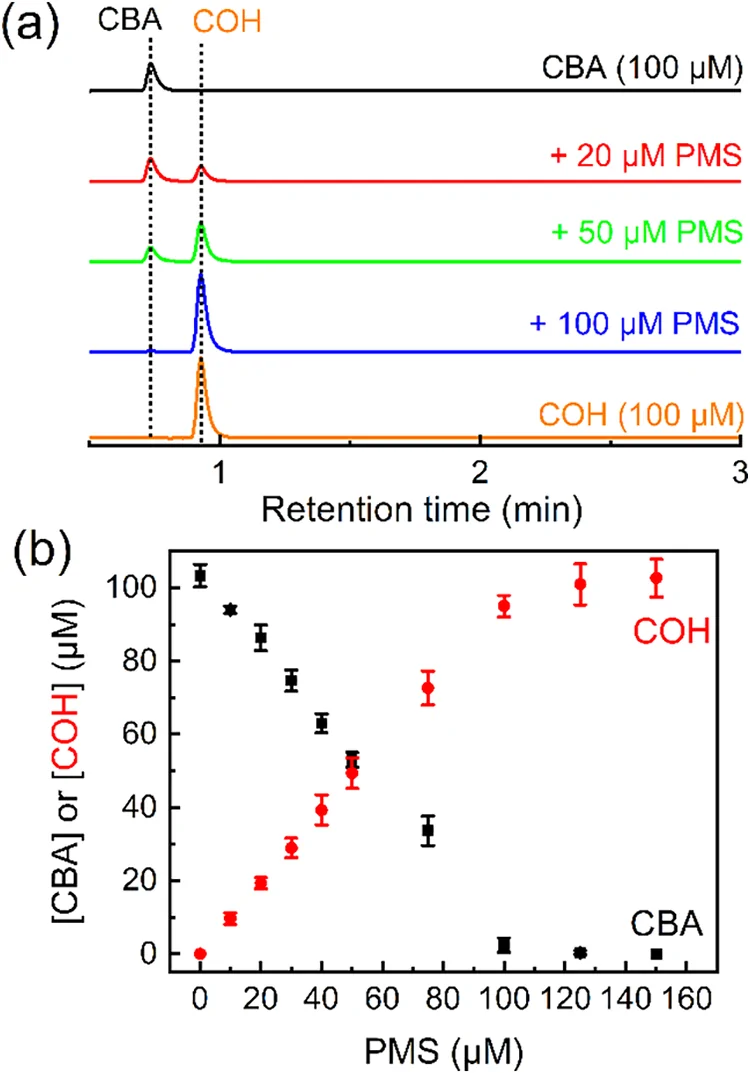
Stoichiometry of the reaction between CBA and PMS. (a) HPLC-PDA analysis of the incubation mixture containing the CBA probe alone and with different concentrations of PMS. (b) Stoichiometric analysis of the reaction between CBA and PMS. The concentrations of CBA and COH calculated from HPLC data were obtained from incubations containing CBA (100 μM), dtpa (100 μM), and PMS (0–150 μM) in phosphate buffer (0.1 M, pH 6.8) with MeCN (0.1%). HPLC traces were recorded at 330 nm.

**Scheme 2:**
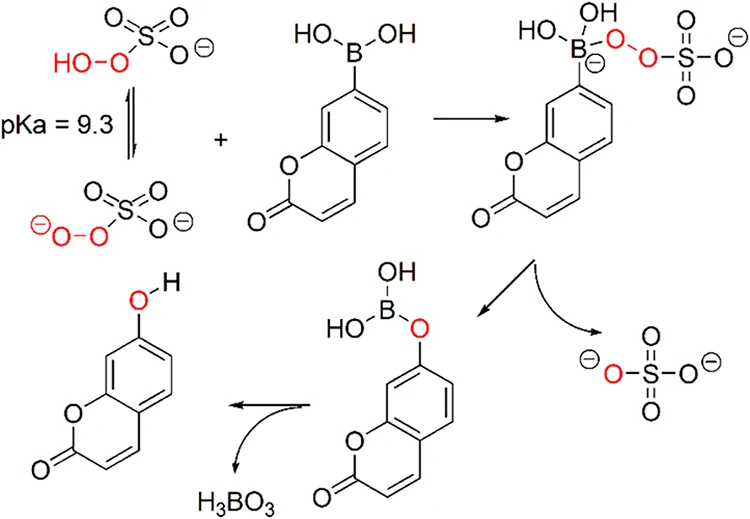
Proposed Reaction Mechanism between Coumarin Boronic Acid and PMS

These results encouraged us to evaluate the sensitivity of the CBA-based assay for determining and quantifying PMS.

Within the PMS concentration range of 0 to 250 nM (Figure 1b), the intensity of the emission band is linearly dependent on the concentration of added peroxymonosulfate (*R* ^2^ = 0.996). Using the signal-to-noise ratio, we determined the LOD to be 20 nM and the LOQ to be 200 nM (see Figure S2). Comparing the obtained values with the available sensitivities of other methods reported in the literature for PMS detection (Table S1), we conclude that, in terms of simplicity and the number of chemicals used, the developed CBA probe-based method is the simplest among those reported.

### Development of the CBA-Based Assay

The efficacy and rapid separation capabilities of HPLC, combined with the sensitivity, selectivity, simplicity, and cost-effectiveness of fluorescence detection, make HPLC with fluorescence detection (HPLC-FLD) an ideal tool for the routine measurement and detection of various analytes. Therefore, the next step in developing the CBA-based assay for PMS detection involved using HPLC-FLD. Fluorometric detection methods that rely only on emission measurements can be misleading because fluorescent dyes formed during the probe’s reaction with the oxidant may also participate in reactions that create fluorescent or nonfluorescent products.^41,48–54^ Results based on HPLC traces recorded with an absorption detector (Figure 2b) show that an excess of PMS (1.5 equiv) did not destroy a coumarin dye, as also evident from the lack of influence of PMS on the COH’s fluorescence intensity (see Figure S3). This is consistent with the literature, which reports that PMS without activation does not oxidize phenols.^55^ Figure 3a shows the HPLC-FLD traces of COH generated from CBA incubated with different amounts of PMS for 5 min at room temperature. With increasing PMS concentration, the HPLC peak intensity due to COH increased (Figure 3a). Within the PMS concentration range of 0 to 50 nM (Figure 3b), the COH HPLC-FLD peak area is linearly dependent on the added PMS concentration. The assay demonstrated good linearity (*R* ^2^ = 0.998), enabling estimation of LOD and LOQ values, based on the signal-to-noise ratio, 5 and 40 nM, respectively. However, even at a PMS concentration of 1 nM, we observed an increase in the COH peak area, as shown in Figure S4. Notably, the LOD and LOQ values obtained with CBA and HPLC-FLD are the lowest among previously reported methods (Table S1). Consequently, the CBA-based HPLC-FLD method appears to be currently the most sensitive analytical technique for measuring PMS in aqueous solutions.

**Figure 3:**
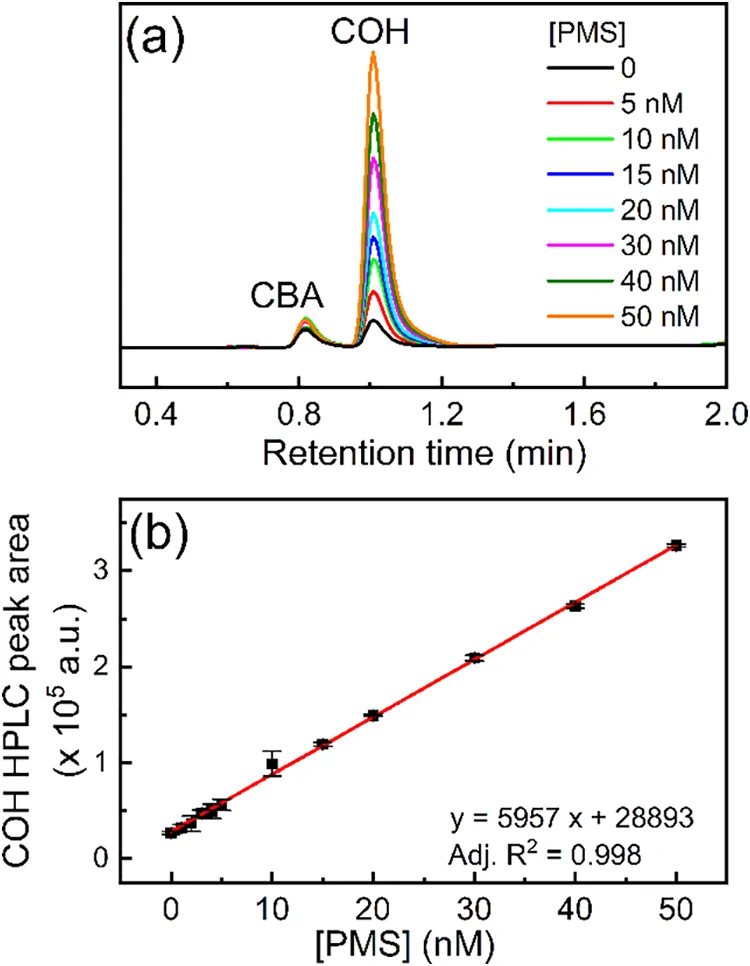
(a) HPLC-FLD chromatograms recorded after adding different concentrations of PMS (5–50 nM) to the CBA probe (20 μM) in the phosphate buffer solution (50 mM, pH 6.8) containing MeCN (0.1%) and dtpa (10 μM). HPLC-FLD traces were recorded after 2 min of incubation at 25 °C and with excitation and emission set at 330 and 450 nm, respectively; (b) The dependence of the peak area of the COH signal on the PMS concentration (1–50 nM). Error bars indicate standard deviations of triplicates.

### Selectivity of the Assay and Anti-Interference Performance in the Presence of Coexisting Peroxide

The selectivity of CBA-based assays for PMS detection was investigated by recording emission spectra and HPLC-FLD chromatograms. First, we investigated whether the CBA probe can distinguish PMS and PDS. After mixing the CBA probe with PMS or PDS, we recorded the chromatograms at 5 and 100 min of incubation at room temperature. HPLC analysis of both reaction mixtures, as shown in Figure S5, indicates that the chromatographic signal originating from COH was detected only in the CBA solution to which PMS was added. Extending the incubation time of the mixture containing CBA and PDS to 100 min does not produce a chromatographic signal corresponding to 7-hydroxycoumarin. After such a long incubation time, we were unable to register the COH signal using either an absorption detector (Figure S5a) or a fluorescence detector (Figure S5b). The CBA’s insensitivity to PDS further supports the proposed mechanism of CBA oxidation by PMS, as shown in Scheme 2. We also compared the fluorescence response of CBA to hydrogen peroxide and assessed the influence of H_2_O_2_ or PDS, as coexisting oxidants, on the efficacy of the PMS-generated fluorescence signal. As shown in Figure 4a, a fluorescence band appears within 2 min after the addition of PMS into a solution of CBA. Neither H_2_O_2_ nor PDS generates a fluorescence signal within such a short time frame.

**Figure 4:**
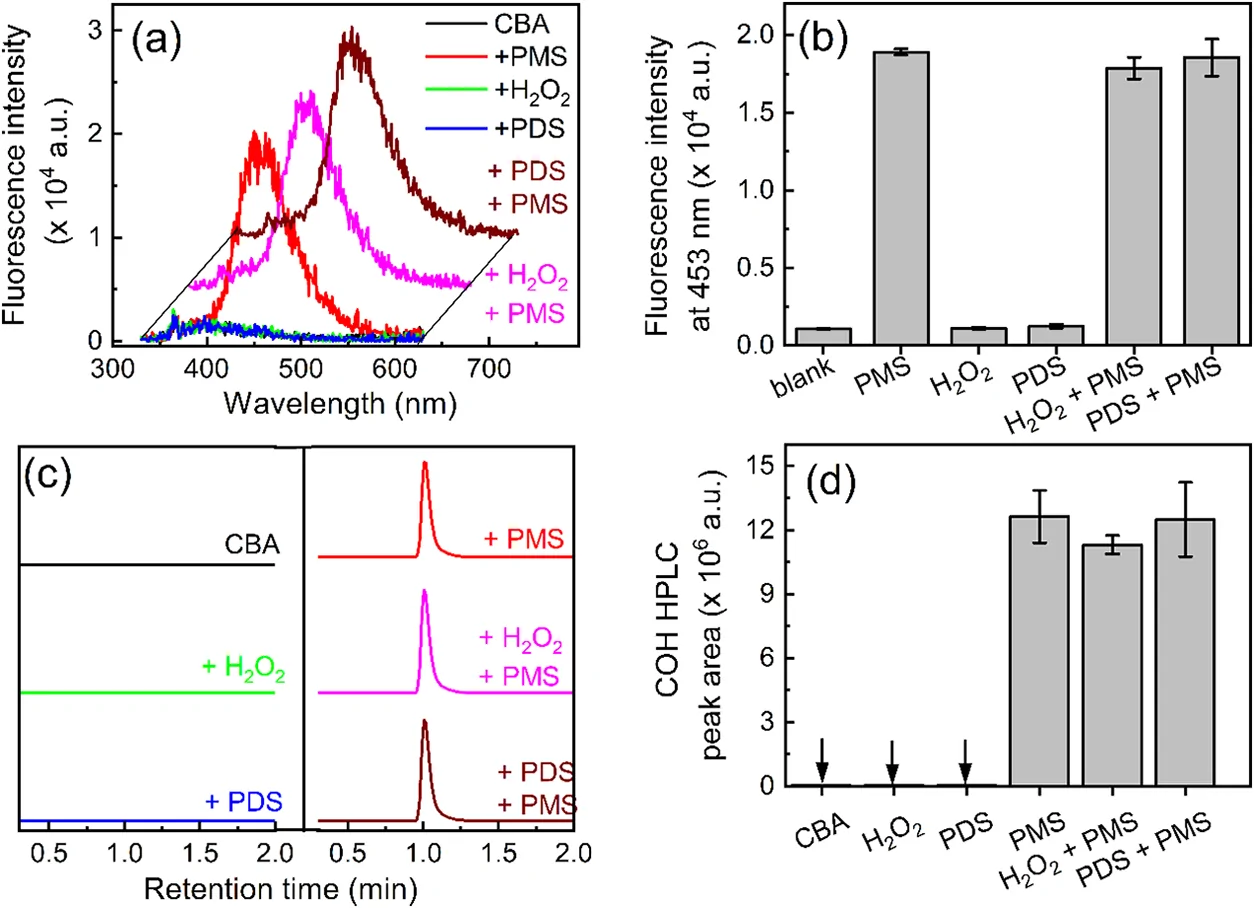
Comparison of the reactivity of CBA toward PMS, PDS, and H_2_O_2_, and the influence of PDS or H_2_O_2_ on the amount of COH generated after the addition of PMS. (a) Fluorescent response of CBA (100 μM) upon addition of oxidants (2 μM) and the influence of PDS (2 μM) or H_2_O_2_ (2 μM) on the determination of PMS (2 μM). Fluorescence spectra were recorded after 2 min of incubation at 25 °C, after a 10-fold dilution of the reaction mixture. (b) Comparison of the maximum fluorescence intensities registered after oxidant addition. Experiments were conducted in phosphate buffer (10 mM, pH 6.8) containing dtpa (10 μM) and MeCN (0.2%; v/v). (c) HPLC-FLD chromatogram traces recorded for the mixture of CBA (20 μM) with oxidants (2 μM) and the influence of PDS (2 μM) or H_2_O_2_ (2 μM) on the determination of PMS (2 μM); (d) Comparison of the amounts of COH in the reaction mixtures. The arrows show the amount of COH in the control solution containing only CBA or after the addition of H_2_O_2_ or PDS. Chromatograms were recorded after 2 min of incubation in a phosphate buffer (10 mM, pH 6.8) containing dtpa (10 μM) and MeCN (0.2%). Error bars indicate standard deviations of triplicates.

The comparison of fluorescence emission intensities (Figure 4b) shows that the addition of PMS, but not H_2_O_2_ or PDS, results in an 18-fold increase in fluorescence intensity. Moreover, adding PMS to a solution that already contains H_2_O_2_ or PDS increases fluorescence intensity to the same level as PMS alone. HPLC-FLD-based analyses of COH (Figure 4c,d) further confirmed those observations. This indicates that the amount of COH generated in the reaction between CBA and PMS is unaffected by the presence of either hydrogen peroxide or PDS in the solution when analyzing the products within 2 min after mixing. It should be emphasized, however, that the practical selectivity of the assay arises from the kinetic window selected for PMS detection, not from the absolute selectivity of CBA for PMS. Still, as incubation time increases, CBA can be oxidized by H_2_O_2_.

### Reaction Kinetics and Effects of Inorganic and Organic Contaminants

Next, we determined the second-order rate constant of the oxidation of CBA by PMS using the stopped-flow technique. Reaction kinetics is a crucial factor controlling the practical use of fluorescent probes for monitoring analytes in complex media. The kinetics of CBA oxidation by PMS was investigated under pseudo-first-order conditions with an excess of the boronate probe. Results presented in Figure S6 show that PMS reacts with CBA at the rate constant of *k*_PMS‑CBA_ = (1.11 ± 0.01) × 10^4^ M^–1^s^–1^. We tentatively attribute the high reactivity of PMS toward CBA compared to other nucleophilic oxidants to the oxidants’ p*K*_a_, which influences the fraction of boronate-reactive anion (α^A^) present in solution at pH 7.4 (see Table S2). Another factor that can affect the kinetics of PMS-induced CBA oxidation is the elimination of a sulfate anion, a good leaving group, whose negative charge is extensively delocalized over the oxygen atoms of the sulfate group, resulting in a weakly basic and highly stabilized anion. The sulfate and carbonate anions have the largest Gibbs free energy of hydratation (Δ*G*_f_^0^), −266.0 ^56^ and −314 kcal mol^–1^,^57^ respectively, compared with other leaving groups, i.e., ^–^OH, Cl^–^, NO_2_^–^, which have Δ*G*_f_^0^ of −104.5,^58^ −74.0,^59^ −69.3 kcal mol^–1^,^60^ respectively, suggesting that the solvation of sulfate and carbonate ions is a factor that determines the kinetics of the oxidation process. Both peroxymonocarbonate and peroxymonosulfate have relatively high second-order rates of CBA oxidation, (1.11 ± 0.01) × 10^4^ and 1.7 × 10^2^ M^–1^s^–1^,^61^ respectively, compared to the fraction of α^A^. Moreover, the results of DFT calculations (Figure S7) indicate that the first two steps of CBA oxidation by PMS occur at lower energy barriers than those for oxidation initiated by H_2_O_2_, by about 4.6 and 6.3 kcal/mol, respectively. The *k*_PMS‑CBA_ value is markedly higher than the direct reaction between PMS and phosphate (*k*_PMS_-_phosphate_ = (2.90 ± 0.20) × 10^–3^ M^–1^s^–1^)^62^ and higher than the self-decomposition of PMS (*k*_PMS_ ∼ 1 × 10^–3^ M^–1^s^–1^)^63^ in solution at a pH of 7. Thus, we can exclude peroxophosphate (O_3_POO^3–^) formed during phosphate oxidation and singlet oxygen produced from the self-decomposition of PMS, as the potential oxidants that generate fluorescent COH in the systems tested. Additionally, the presence of radical scavengers, such as MeOH, 2-PrOH, or *tert*-BuOH, and phenol, a typical organic pollutant, does not affect the fluorescence signal intensity recorded during PMS-induced oxidation of CBA (Figure S8a,b). MeOH and *tert*-BuOH react with PMS with a negligible reaction rate constant (1 × 10^–4^ M^–1^ s^–1^),^55^ compared to their strong reactivity toward both SO_4_^•–^ and ^•^OH. Considering the known *k* values of the reaction between MeOH and SO_4_^•–^ (1.1 × 10^7^ M^–1^ s^–1^)^55^ or ^•^OH (1.1 × 10^9^ M^–1^ s^–1^),^55^ between *tert*-BuOH and SO_4_^•–^ (8.0 × 10^5^ M^–1^ s^–1^)^55^ or ^•^OH (4.2–7.6 × 10^8^ M^–1^ s^–1^),^55,64^ and between 2-PrOH and ^•^OH (2.0 × 10^9^ M^–1^ s^–1^)^65^ or SO_4_^•–^(7.9–8.6 × 10^7^ M^–1^ s^–1^)^66^ one can conclude that SO_4_^•–^ and ^•^OH radicals are not responsible for PMS-induced CBA oxidation. Similarly, phenol, due to its low reaction rate (*k*_PMS‑PhOH_ = (4.7 ± 1.2) × 10^–2^ M^–1^ s^–1^),^55^ is unable to compete with CBA for PMS.

Next, we tested the response of the CBA-based assay in solutions containing some common anions (Cl^–^, HCO_3_^–^, SO_4_^2–^, NO_3_^–^, NO_2_^–^) and cations (K^+^, Mg^2+^, Ca^2+^, Al^3+^) that may coexist with PMS in real-life water samples. Results (Figure 5a,b) indicate that the intensity of the fluorescence emission signal is insensitive to the presence of tested ions. These results align with the existing kinetic data. It is known that PMS oxidizes chlorides and nitrites with a second-order rate constant k_PMS‑Cl_- = (1.4–2.5) × 10^–3^ M^–1^ s^–1^ ^47,67^ and k_PMS‑NO3_- = 3.1 × 10^–1^ M^–1^ s^–1^,^68,69^ whose values are 5–7 orders of magnitude lower than those of the oxidation reaction of CBA, making them unable to interfere with the detection of PMS using the developed pro-fluorescent test.

**Figure 5:**
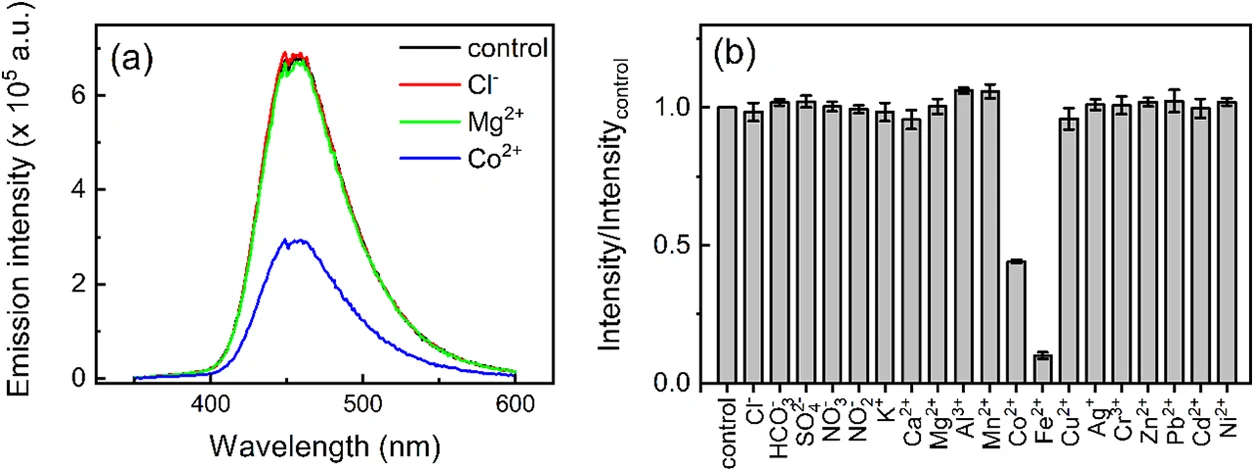
Effect of additives on the fluorescent response of CBA toward PMS. (a) Representative fluorescence spectra of the CBA probe (100 μM) after its oxidation by PMS (60 μM) under different conditions, in a phosphate buffer (pH 6.8, control) and with the addition of Cl^–^ (0.5 mM), Mg^2+^ (0.1 mM), or Co^2+^(0.5 μM). Spectra were recorded after a 10-fold dilution of the reaction mixture. (b) Effects of inorganic anions ([Cl^–^] = 0.5 mM; [HCO_3_^–^] = 10 μM; [SO_4_^2–^] = 50 μM; [NO_3_^–^] = [NO_2_^–^] = 25 μM) and cations ([K^+^] = 0.5 mM, [Ca^2+^] = [Mg^2+^] = 0.1 mM, [Al^3+^] = [Mn^2+^] = 6 μM; [Cu^2+^] = 3 μM; [Co^2+^] = [Fe^2+^] = [Ag^+^] = [Cr^3+^] = [Zn^2+^] = [Ni^2+^] = 0.5 μM; [Pb^2+^] = [Cd^2+^] = 0.25 μM) on the fluorescent response of CBA toward PMS. Experiments were conducted in phosphate buffer (pH 6.8, 0.05 M) with dtpa (10 μM) and DMSO (0.1%). Error bars indicate standard deviations of triplicates.

The effects of bicarbonates on PMS-induced degradation of organic contaminants have been extensively studied. It was previously reported that HCO_3_^–^ accelerates the decomposition of PMS;^63^ however, the second-order rate constant of this process was not determined. Based on the results presented in published literature,^63^ it can be estimated that the *t*_1/2_ of this process is approximately 100 min; therefore, the rate constant of the reaction of PMS with HCO_3_^–^ to form peroxymonocarbonate (HCO_4_^–^) is expected to be much slower than the determined value for k_CBA‑PMS_. Figure S9 shows a comparison of the kinetics profiles of COH formation during the oxidation of the CBA probe by PMS, H_2_O_2_, and HCO_4_^–^ formed during the reaction of H_2_O_2_ with NaHCO_3._ The results indicate that COH formation is fastest in the PMS-containing solution compared with the oxidation of CBA initiated by the other oxidants tested.

Multiple reports describe the use of PMS in homogeneous systems with metal cations to remove organic contaminants. Therefore, we also investigated whether water-soluble metal salts can disrupt the reaction between CBA and PMS. Figure 5b shows that, apart from Co^2+^ and Fe^2+^, the remaining heavy metal cations did not compete effectively for PMS under the measurement conditions and therefore did not prevent the formation of fluorescent COH. For Co^2+^ and Fe^2+^, reductions in fluorescence intensity by 65% and 91%, respectively, were observed. Previous reports indicate that cobalt­(II)^6^ and iron­(II)^70^ salts effectively activate PMS, so it is not surprising that their presence in the solution efficiently decomposes PMS and prevents its reaction with CBA. The reported *k*_PMS‑Fe2+_ values range from 3.7 × 10^4^ to 4.45 × 10^5^ M^–1^s^–1^.^70^ In contrast, the *k* value of PMS with Co^2+^ was estimated as 100 M^–1^ s^–1^.^71^ However, it is suggested that the regeneration of Co­(II) through the reaction of Co­(III) with PMS is a crucial step in the effectiveness of the Co^2+^ salt as a PMS activator.^71–73^ This shows that an assay that utilizes the oxidative release of fluorescent coumarin from the CBA probe can detect residual PMS remaining in solution after AOP processes. During metal (M^n+^)-induced PMS activation, several reactive species, both radical and nonradical, have been identified or proposed to contribute to the degradation of organic pollutants. However, the proposed oxidation mechanisms are controversial, and ROS detection methods ideally require multiple probe combinations.^74^ The CBA-based test does not define which ROS are formed in the reaction of PMS with M^n+^, but based on competition kinetics it allows for the determination of the most effective PMS activators.

### Detection of PMS in Real Water Samples

We tested the suitability of the CBA-based assay to determine PMS in real-life samples by detecting PMS in water samples collected from the Grabia River and the Jana ponds, as well as in wastewater from the polyester fabric dyeing process at the Bilinski Factory of Color. Results of these experiments are presented in Table 1. Recoveries of 93–110% for spiked PMS at concentrations of 400–1200 nM were obtained for all tested samples, indicating that the measured concentrations were statistically close to the added values. Also, RSDs below 5% indicate good precision of the CBA-based HPLC-FLD method.

**Table 1:** PMS Detection in Spiked Water Samples

Sample	Spiked (nM)	Found (nM)	Recovery (%)	RSD (*n* = 3; %)
Tap water	400	415	103.7	2.5
	750	776	103.5	0.7
	1200	1274	106.2	0.5
Jan Ponds	400	396	99.0	4.7
	750	724	96.5	0.8
	1200	1243	103.6	0.2
Grabia River	400	417	104.3	0.4
	750	752	100.3	1.3
	1200	1295	107.9	1.4
Dyes wastewater	400	362	90.5	4.5
	750	765	102.0	2.1
	1200	1323	110.2	2.9

### Detection of PMS Formed *In Situ* in Cell-Free and Cellular Systems

Autoxidation of sulfites in chemical and biological systems generates radicals that participate in chain reactions. PMS is one of the products reported to be formed in this complex system (Scheme 1), but it has never been quantified. Thus, we decided to test whether the CBA probe could detect PMS in an aerobic buffer containing NaHSO_3_. A comparison of the dynamics of COH formation during the oxidation of the CBA probe by PMS, H_2_O_2_, and in the presence of NaHSO_3_ is shown in Figure 6a. The kinetic profiles show that in bisulfite solution, COH is formed at a rate higher than that of CBA oxidation by H_2_O_2_. Moreover, catalase (CAT) did not affect the rate of fluorescent signal build-up, either during the conversion of CBA initiated by the bolus addition of PMS or during PMS formation *in situ* during (bi)­sulfite autoxidation, excluding the possibility of H_2_O_2_ involvement. H_2_O_2_ oxidizes boronic acids with a second-order rate constant of ∼1 M^–1^ s^–1^ ^41^, 4 orders of magnitude lower than the rate constant we determined for the reaction between CBA and PMS. Thus, we can conclude that hydrogen peroxide is not responsible for the generation of COH in the sulfite solution containing CBA. Next, we incubated the CBA probe in phosphate buffer containing varying amounts of NaHSO_3_ and measured the increase in fluorescence over 1 h. Parallel samples were analyzed by HPLC. The results of these experiments indicate that CBA is converted to a fluorescent COH during the autoxidation of HSO_3_^–^/SO_3_^2–^ in an air-equilibrated aqueous solution, and that the rate of COH formation is proportional to the NaHSO_3_ concentration (Figure 6b,c).

**Figure 6:**
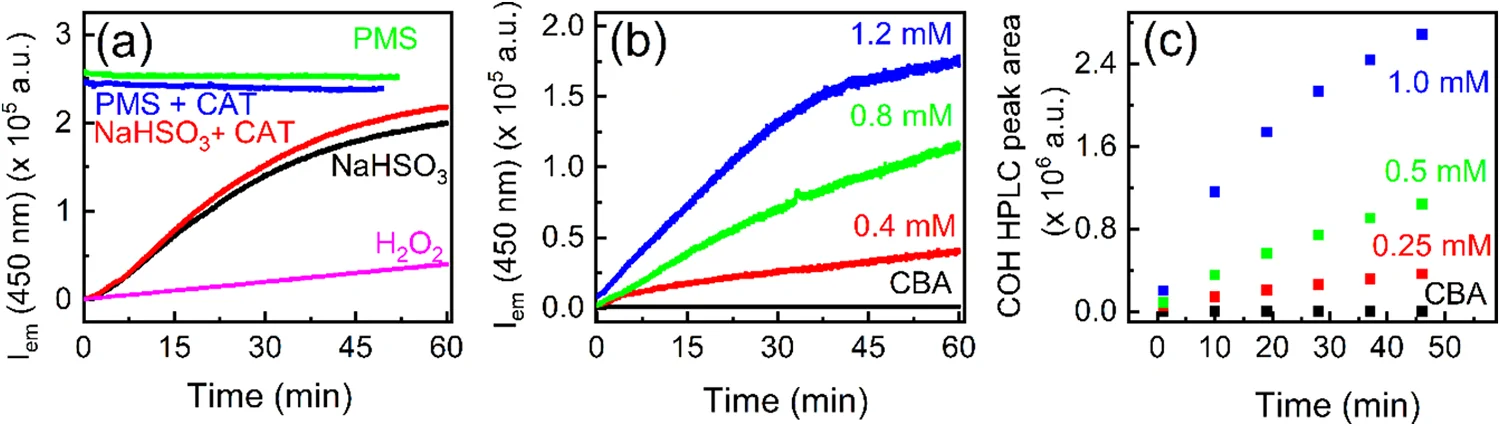
(a) Kinetic profiles of COH formation in the air-equilibrated phosphate buffer containing CBA (10 μM) and NaHSO_3_ (200 μM) with and without the addition of catalase (CAT, 100 U/ml). For comparison, the kinetic profile of CBA oxidation by H_2_O_2_ (200 μM) and PMS (100 μM), alone or with CAT (100 U/mL), was measured; (b) Representative kinetics profile of COH formation in the air-equilibrium water solution containing CBA (10 μM), phosphate buffer (0.1 M, pH 6.8), dtpa (100 μM), and different concentrations of NaHSO_3_ (0–1.2 mM); Emission profiles were collected using excitation at 325 nm. (c) The dependence of the HPLC peak area of COH on the concentration of NaHSO_3_. HPLC-FLD traces recorded during the incubation of CBA (15 μM) in the air-equilibrated water containing phosphate buffer (0.05 M, pH 6.8), dtpa (50 μM), and bisulfite (0–1 mM).

To further gain insights into the CBA oxidation mechanism during (bi)­sulfite autoxidation, we tested the effects of different chemical/enzymatic scavengers, as shown in Figure 7. First, we tested the role of radical intermediates formed during autoxidation of HSO_3_^–^/SO_3_^2–^. The addition of the DMPO (5,5-dimethyl-1-pyrroline-*N*-oxide) spin trap caused inhibition of COH formation, which can be attributed to the quenching of radical intermediates and the interruption of the chain reactions (Scheme 1). This confirms that the oxidant responsible for CBA oxidation is generated during the radical-initiated process. Earlier reports indicate that superoxide dismutase (SOD) acts as an enzymatic initiator of sulfite autoxidation.^23^ When SOD was present in the solution with CBA and bisulfite, it increased the amount of COH produced. Additionally, in the solution containing both SOD and catalase (CAT), COH yield was the same; thus, contribution of CBA oxidation by H_2_O_2_ to the product formation was excluded. We thus confirmed that the pro-fluorescent probe-based test can detect PMS generated during SOD-amplified oxidation of sulfite. We also confirmed that CBA is not oxidized by peroxydisulfate (PDS), as the 30 min incubation of CBA with PDS in the solution at 80 °C did not produce COH, which further supports our results above (Figure 4) and indicates that CBA oxidation is not initiated by SO_4_^•–^ radicals. Similarly, the presence of CAT had no effect on the amount of COH formed in the CBA and NaHSO_3_ reaction mixture (Figure 7). This confirms that the oxidation of CBA in aqueous sulfite solutions is not due to the action of H_2_O_2_.

**Figure 7:**
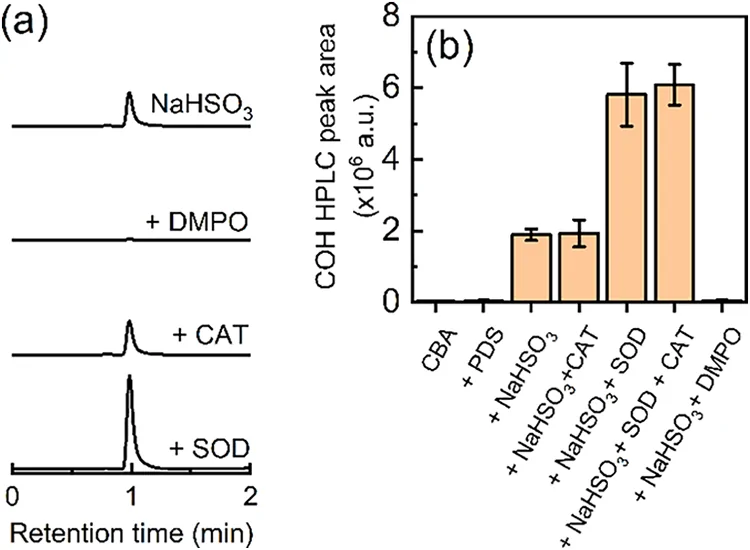
Oxidation of CBA to COH by PMS that was generated in chemical and enzymatic systems containing NaHSO_3_. (a) HPLC-FLD traces recorded after 30 min of incubation of the solutions containing CBA (15 μM) and NaHSO_3_ (1 mM) alone or in the presence of DMPO (20 mM) or enzymes - SOD (0.01 mg/mL) or CAT (0.01 mg/mL); (b) Effect of the different additives on the formation of COH after 30 min of incubation. Error bars indicate standard deviations of triplicates.

Finally, we investigated whether a developed CBA-based assay could be used to detect and quantify PMS in RAW 264.7 cell culture. *In vitro* data indicate that sulfite has adverse effects on nerve and other cell types.^75^ The reported lowest-observed-adverse-effect concentration (LOAEC) of sulfite (expressed as SO_2_ concentration) differed significantly (from 5 μM to 4 mM), and an increase in ROS levels was observed without their identification.^75^ Therefore, we first measured the effect of bisulfite concentration (10, 100, 1000 μM) on macrophage cell viability. Results presented in Figure S10 show that at a sulfite concentration of 1 mM, the viability of RAW 264.7 murine macrophage cells, as measured by an ATP-sensitive luminescent assay, is reduced to 80%. Therefore, we measured peroxymonosulfate production in macrophages incubated with PMA and treated with two bisulfite concentrations (10 and 100 μM), which did not affect cell viability. PMA stimulates macrophages to produce O_2_^•–^, which next reacts with SO_3_^2–^ (*k* = 82 M^–1^s^–1^)^17^ and drives the radical chain reaction of sulfite oxidation. Activation of macrophages with PMA in the presence of 10 μM or 100 μM of NaHSO_3_ resulted in increased COH fluorescent signal (Figure 8a), consistent with PMS formation. As catalase (CAT) does not affect the rate of COH formation, H_2_O_2_ does not seem to contribute to boronate oxidation under the conditions used, either directly or *via* oxidation of NaHSO_3_ to PMS. It is also evident that SOD promotes the oxidation of bisulfite, resulting in greater initial amounts of COH formed, similar to a cell-free system (Figure 7). At the same time, the rate of CBA oxidation over time was to some extent decreased by SOD, suggesting the involvement of O_2_^•–^ in the propagation of PMS formation. Biological production of HOCl requires the enzyme myeloperoxidase (MPO) and H_2_O_2_,^76^ but RAW macrophages express quite low levels of MPO.^77,78^ Moreover, high reactivity of HOCl toward cellular thiols and amines makes rather unlikely to oxidize boronic acids. To exclude the participation of other oxidants, e.g., HOCl or peroxynitrite, in the reaction with CBA, it is necessary to perform appropriate control experiments, modulating their production (e.g., enzyme inhibitors) and/or their availability chemical or enzymatic scavengers. For example, the participation of HOCl in the oxidation of CBA can be ruled out by exploiting HOCl’s high reactivity toward thiols. As was shown in Figure S11, in the presence of GSH, the oxidation of CBA by HOCl was completely inhibited, whereas GSH had no effect on oxidation induced by PMS and peroxynitrite. Additionally, a major advantage of boronate probes, as noted in our previous investigations,^41,48–54^ is the formation of HOCl- and peroxynitrite-specific products that can be detected by HPLC. The detection of a chlorinated or nitrated fluorophore confirms the participation of HOCl or peroxynitrite, respectively, in the oxidation of the boronate probe. Our chromatograms in Figure S12 exclude the involvement of peroxynitrite in the oxidation of CBA, as evidenced by the absence of the peroxynitrite-specific products, including 7-nitrocoumarin. Moreover, to exclude the participation of HOCl and/or ONOO^–^ in the formation of COH in RAW 264.7 cells, we performed UPLC analysis of cultured media, which confirms that COH is the sole product formed during oxidation of CBA (Figures 8c and S13), and its yield is not decreased by the addition of CAT, eliminating the possible involvement of H_2_O_2_ in probe oxidation. Therefore, we propose that detailed HPLC-based profiling of the oxidation products, together with pharmacological and/or genetic manipulation approaches will enable identification of the oxidant(s) involved. These results provide strong evidence supporting the formation of PMS in the cellular system and demonstrate the feasibility of monitoring its production in real time using fluorogenic boronate probes.

**Figure 8:**
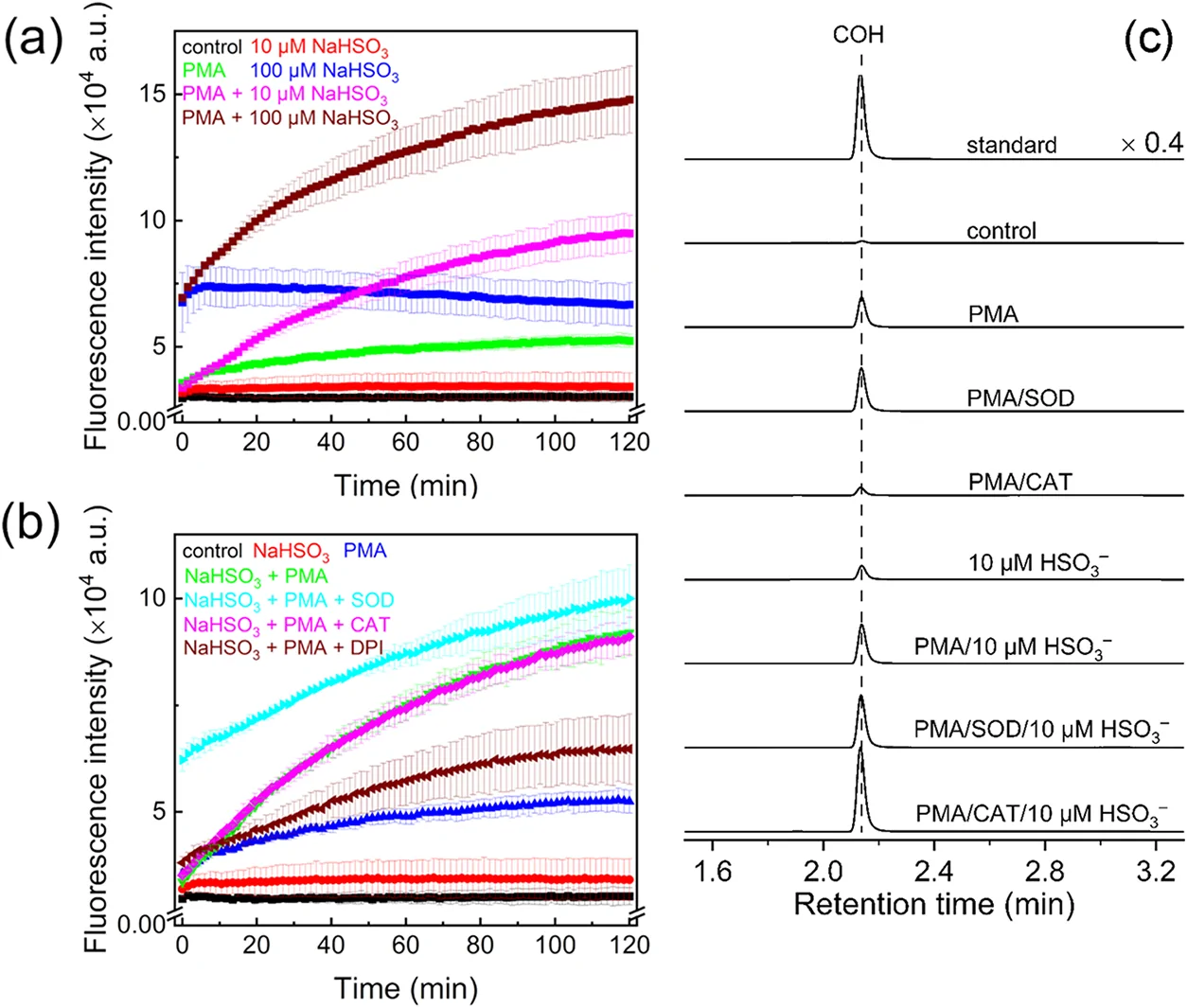
Fluorescent detection of PMS in RAW 264.7 cells. (a) Representative kinetic traces of CBA (20 μM) oxidation in RAW 264.7 macrophages in DPBS-G and treated with PMA (1 μM) in the presence or absence of NaHSO_3_ (10 or 100 μM); (b) Parallel experiments evaluating the inhibitors’ effects on the CBA oxidation in PMA-activated macrophages in the presence of NaHSO_3_ (10 μM). Experimental conditions were the same as in panel (a). The following inhibitors were used: CAT (1 kU/mL), DPI (1 μM), and SOD (0.1 mg/mL). Fluorescence parameters: λ_ex_ = 350 nm, λ_em_ = 460 nm. Data are presented as mean ± SD of three independent replicates. (c) UPLC traces obtained from the culture media of RAW 264.7 cells incubated in the presence or absence of CBA (20 μM), PMA (1 μM), SOD (0.1 mg/mL), catalase (1 kU/mL) and bisulfite (10 μM). The concentration of the COH standard was 1 μM. The UPLC traces were recorded using fluorescence detector (λ_ex_ = 332 nm and λ_em_ = 450 nm).

### Limitations and Future Directions

Arylboronate-based probes are commonly used in redox biology research to detect biologically relevant two-electron oxidants, primarily hydrogen peroxide and peroxynitrite (ONOO^–^). Due to the 10^4^ times higher rate constant of the CBA reaction with PMS than with H_2_O_2_, the developed PMS detection method is practically insensitive to H_2_O_2_ when low probe concentrations and short incubation times (e.g., 2 min) are used. This may be taken advantage of for end-point PMS measurements. Under such conditions, peroxynitrite-mediated oxidation may also be excluded due to the short half-life of ONOO^–^ at pH 6.8 (<2 s).^41^ To exclude the possibility of oxidation by HOCl,^41,49,50,52,79^ the addition of an equimolar amount (corresponding to the boronate probe) of thiol (e.g., GSH) may be used to quench HOCl (see Figure S11). In cellular systems, where long-term monitoring is used, H_2_O_2_ and other nucleophilic two-electron oxidants (e.g., peroxymonocarbonate, amino acid and protein hydroperoxides, peroxynitrite) may contribute to probe oxidation, as demonstrated in multiple publications.^41,80^ In such cases, the absolute identification of the oxidant may not be trivial. The requirement of (bi)­sulfite, and the lack of inhibitory effects of H_2_O_2_ scavengers (e.g., catalase) may suggest the involvement of PMS. Peroxynitrite reacts with arylboronic probes with a rate constant value 100 times higher (^*2*^ *k* ∼ 1 × 10^6^ M^–1^s^–1^)^41^ than of PMS. However, in the minor radical pathway of this reaction, peroxynitrite-specific products, i.e., reduced and nitrated derivatives of probes, are formed. Unlike ONOO^–^, PMS does not produce minor products during its reaction with CBA. Thus, the HPLC detection of an ONOO^–^-specific product in the analyzed solution indicates that ONOO^–^ was involved in the oxidation of arylboronate and may allow for distinguishing ONOO^–^ and PMS, as we showed during analysis of the reaction mixture of CBA with PMS and ONOO^–^ (see Figure S12). Additional approaches to distinguish different oxidants of boronate-based probes in complex biological systems should be developed. Since the LOD and LOQ depend on the fluorescence quantum yield (ϕ_em_) and the brightness of the dyes formed in the reaction with PMS, we believe that a boronate probe based on a hydroxyfluorophore with a higher ϕ_em_ and lower p*K*_a_ can improve the detection of PMS in cells. Moreover, to minimize biological interference from the UV excitation light used during the experiment with CBA, a probe that absorbs and emits in the visible or NIR range should be beneficial for biological samples. Another feature that enables PMS detection in specified organelles is the probe’s conjugation to the organelle-targeting group. The CBA-based assay for PMS is sensitive to Fe^2+^ and Co^2+^, cations commonly used in AOP wastewater treatment. This behavior opens a new approach for initial screening in the development of novel metal-catalyzed PMS oxidation processes.

## Conclusions

In this paper, we present a simple, fast, and sensitive fluorescence-based assay for the detection of PMS in chemical, enzymatic, and cellular systems. We have developed two methods based on 7-hydroxycoumarin (COH, also known as umbelliferone) released after a fast and stoichiometric reaction between PMS and coumarin boronic acid. A simple spectrofluorometer can detect PMS with a limit of detection of 200 nM or lower, depending on the instrumental settings. In terms of simplicity and the number of chemicals used, the developed CBA-based fluorometry method is the most straightforward assay among those reported in the literature. Combining the CBA-assay with HPLC-FLD detection results in ultrasensitive detection of PMS, with a detection limit of 5 nM and a quantification limit of 40 nM. The protocol we developed is the most sensitive of the reported methods for detecting PMS and shows good selectivity (among PMS, PDS, and H_2_O_2_). Due to the above advantages, the developed assay carries promising potential for practical applications in both end-point and real-time monitoring of PMS generation, not only in chemical but also in enzymatic systems and in cell culture, as demonstrated in the SOD-based enzymatic system and in RAW 264.7 murine macrophage cells, respectively. However, rigorous identification of the oxidant(s) involved requires appropriate controls, pharmacologic and/or genetic cell manipulation, in combination with HPLC/UPLC profiling of the products formed.

## Supplementary Material



## Notes

Comparing a CBA-based PMS detection method with other PMS detection methods (Table S1); comparing of the properties of PMS with other nucleophilic oxidants known to react with arylboronates (Table S2); figures showing the impact of solution pH on the yield of COH, estimation of LOD by fluorimetry, stability of COH in the presence of PMS, CBA reactivity toward PMS and PDS, determination of second-order rate constant of the reaction between CBA and PMS, and kinetic profiles of COH formation during the oxidation of CBA by PMS, H_2_O_2_, and H_2_O_2_ in the presence of NaHCO_3_, results of DFT calculations (PDF)
