# Supplementary material for: Simple, Fast, and Ultrasensitive Fluorescent Detection of Peroxymonosulfate in Chemical, Enzymatic, and Cellular Systems
Source: Anal Chem. 2026 Sep 22;98(41):29541–52. doi: 10.1021/acs.analchem.6c03097 (PMC13689399; doi:10.1021/acs.analchem.6c03097)
Supplement: Supplementary file 1 [file ac6c03097_si_001.pdf]

Supporting Information

Simple, Fast, and Ultrasensitive Fluorescent Detection of Peroxy-monosulfate in Chemical, Enzymatic, and Cellular Systems

Radosław Podsiadły[1*], Aleksandra Grzelakowska[1], Kumari Neelam[1], Radosław Michalski[2], Michał Rostkowski[2], Renata Smulik-Izydorczyk[2], Adam Sikora[2], Jacek Zielonka[3]

[1] Institute of Polymer and Dye Technology, Faculty of Chemistry, Lodz University of Technology, Stefanowskiego 16, 90-537 Lodz, Poland

[2] Institute of Applied Radiation Chemistry, Faculty of Chemistry, Lodz University of Technology, Zeromskiego 116, 90-924 Lodz, Poland

[3] Department of Biophysics, and Redox & Bioenergetics Core, Cancer Center Translational Metabolomics Shared Resource, Medical College of Wisconsin, 8701 Watertown Plank Road, Milwaukee, WI, 53226, United States

* Corresponding author (e-mail: radoslaw.podsiadly@p.lodz.pl)

Table of Contents

Table S1. Comparison between a CBA-based and other PMS detection methods. ……………………………. S3

Figure S1. Comparison of the amount of 7-hydroxycoumarin (COH) formed due to the reaction of the CBA probe with PMS in an aqueous environment at different pH. ……………………………………………………………… S4

Figure S2. Fluorescence response of CBA (100 μM) after addition of PMS (0.25 - 2 μM). ………………………… S4

Figure S3. Stability of COH in the presence of PMS. ………………………………………………………………… S5

Figure S4. (a) HPLC-FLD chromatograms recorded after adding different concentrations of PMS (1-5 nM) to the CBA probe (20 μM) in the phosphate buffer solution (50 mM, pH 6.8) containing MeCN (0.1%) and dtpa (10 μM). HPLC-FLD traces were recorded after 2 minutes of incubation at 25 °C and with excitation and emission set at 330 nm and 450 nm, respectively; (b) The dependence of the peak area of the COH signal on the PMS concentration (1-5 nM). Error bars indicate standard deviations of triplicates. ……………………………..... S5

Figure S5. Comparison of **CBA** reactivity toward peroxymonosulfate (PMS) and peroxydisulfate (PDS). …. S6

Figure S6. Kinetics of the reaction between CBA and PMS. ...……………………………………………….. S6

Scheme S1. General mechanism of phenylboronic acid oxidation induced by anionic peroxides and the anion of hypochlorous acid ……………………………………………………………………………………… S7

Table S2. Comparison of the properties of PMS with other peroxy-oxidants. ...……………………………… S7

Figure S7. Results of DFT calculation. ……………………………………………………………………….. S8

Figure S8. Effect of additives on the fluorescent response of CBA toward PMS. …………………………… S9

Figure S9. Kinetic profiles of COH formation during the oxidation of CBA (10 μM) by PMS (75 μM), H_2O_2 (200 μM), and H_2O_2 (200 μM) in the presence of $NaHCO_3$ (200 μM). ……………………………………………. S9

Figure S10. Effect of $NaHSO_3$, in the presence or absence of PMA and CAT, on the metabolic activity of RAW 264.7 cells. ………………………………………………………………………………………………... S9

Figure S11. Effect of GSH on the oxidation of the CBA probe by HOCl, PMS, and peroxynitrite. ……….. S10

Figure S12. Comparison of the product formed during the reaction of the CBA probe with PMS and peroxynitrite. ……………………………………………………………………………………………….. S11

Figure S13. UPLC detection of PMS in RAW 264.7 cells. ………………………………………………… S12

DFT calculation ..………………………………………………………………………………………… S13

References ..…………………………………………………………………………………………….... S9

Table S1. Comparison between a CBA-based and other PMS detection methods.

Method	Linear range (µM)	LOD (nM)	pH range	Sensitivity to PDS / H_2O_2	Mode of detection, chemicals, and optimal pH	Ref.
Thermometric titrimetry	UNR	50000	UNR	PDS – Yes<br>H_2O_2 - Yes	H_2SO_4 (0.5 or 5 M)	1
HPLC	UNR	500	UNR		0	2
Chemiluminescence	0.4 – 2	50	UNR	PDS – NO<br>H_2O_2 - NO	Turn on; Luminol (30 µM), NaOH (0.2M)	3
KI spectrophotometry	12 - 115	3620	2.5 - 11	UNR	Turn on, KI (100 g/L), $NaHCO_3$ (5 g/L), pH 3	4
DPD spectrophotometry	10 - 100	10000	5.8, 7.0, 8.0	PDS – Yes<br>H_2O_2 - NO	Turn on, *N*,*N*-diethyl-*p*-phenylenediamine (2.5 mM), phosphate buffer (50 mM, pH 7)	5
MO spectrophotometry	0.2 - 100	90	2.0 – 9.0	UNR	Turn off, Methyl Orange (77.6 µM), $CoSO_4$ (2 mM), pH = 5	6
AO7 spectrophotometry	0 - 80	80	2.0 – 8.0	UNR	Turn off, Acid Orange 7 (113.6 µM),$CoSO_4$ (1 mM), pH = 5	7
MB spectrophotometry	0 - 100	100	2.0 – 8.0	UNR	Turn off, Methylene Blue (29.6 µM),$CoSO_4$ (1 mM), pH = 5	7
MV spectrophotometry	0 - 80	80	2.0 – 8.0	UNR	Turn off, Methyl violet (62.4 µM),$CoSO_4$ (1 mM), pH = 5	7
RhB spectrophotometry	0 - 40	40	2.0 – 8.0	UNR	Turn off, Rhodamine B (19.2 µM),$CoSO_4$ (1 mM), pH = 5	7
BA fluorometry	0 - 100	10	5.5	PDS – NO<br>H_2O_2 - NO	Turn on; Benzoic acid (20 mM), $CoSO_4$ (50 µM), pH = 5.5	8
CBA fluorometry	0 - 6	20	6.8	PDS – NO<br>H_2O_2 – NO *	Turn on, Coumarin boronic acid (10 µM), (pH 6.8)	this work
CBA HPLC-FLD	0 - 100	5	4.6 – 10.6	PDS – NO<br>H_2O_2 – NO *	Turn on, Coumarin boronic acid (20 µM), (pH 6.8)	this work

UNR-unreported; * second-order reaction rate constant between CBA and H_2O_2 indicates that 24 h incubation is required for complete oxidation of CBA by H_2O_2.

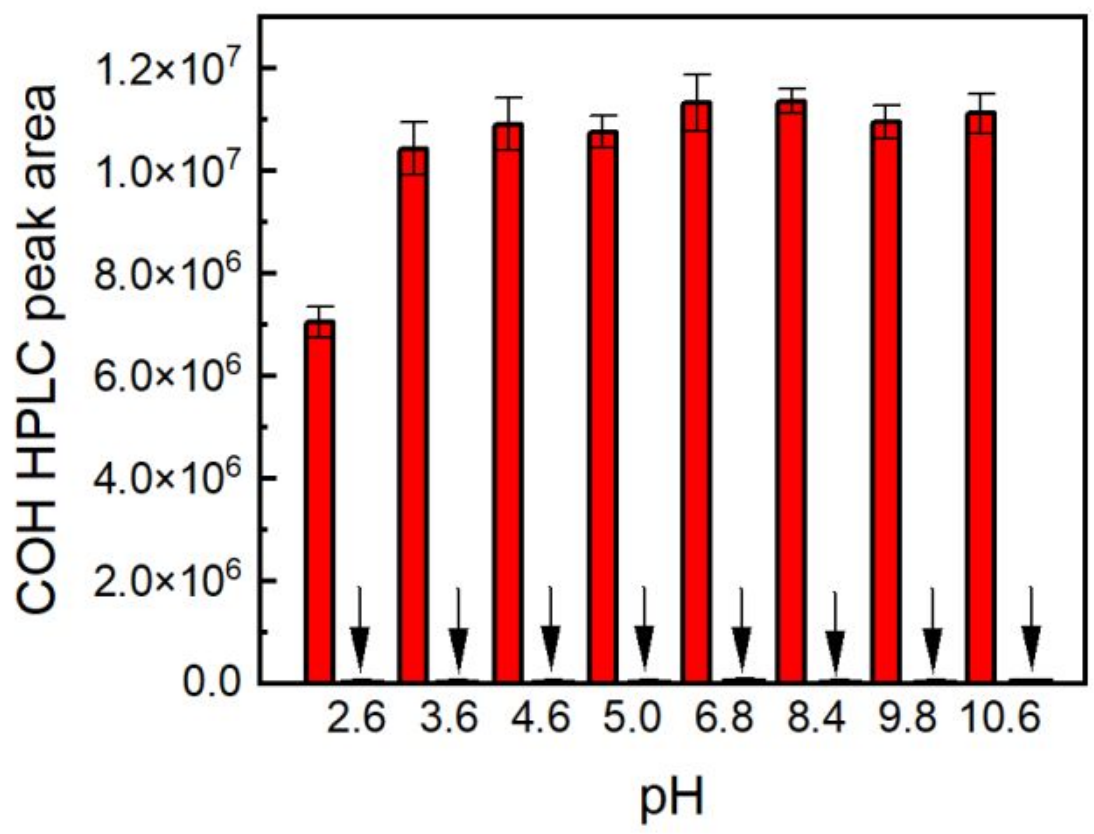

Figure S1. Comparison of the amount of 7-hydroxycoumarin (COH) formed due to the reaction of the CBA probe with PMS in an aqueous environment at different pH. HPLC analysis was performed 5 minutes after adding PMS (2 μM) to the buffer solution containing CBA (10 μM). HPLC traces were recorded with fluorescence detection (excitation, 330 nm; emission, 450 nm). The arrows indicate the amount of COH in the control solution, which contains only CBA. Error bars indicate standard deviations of triplicates.

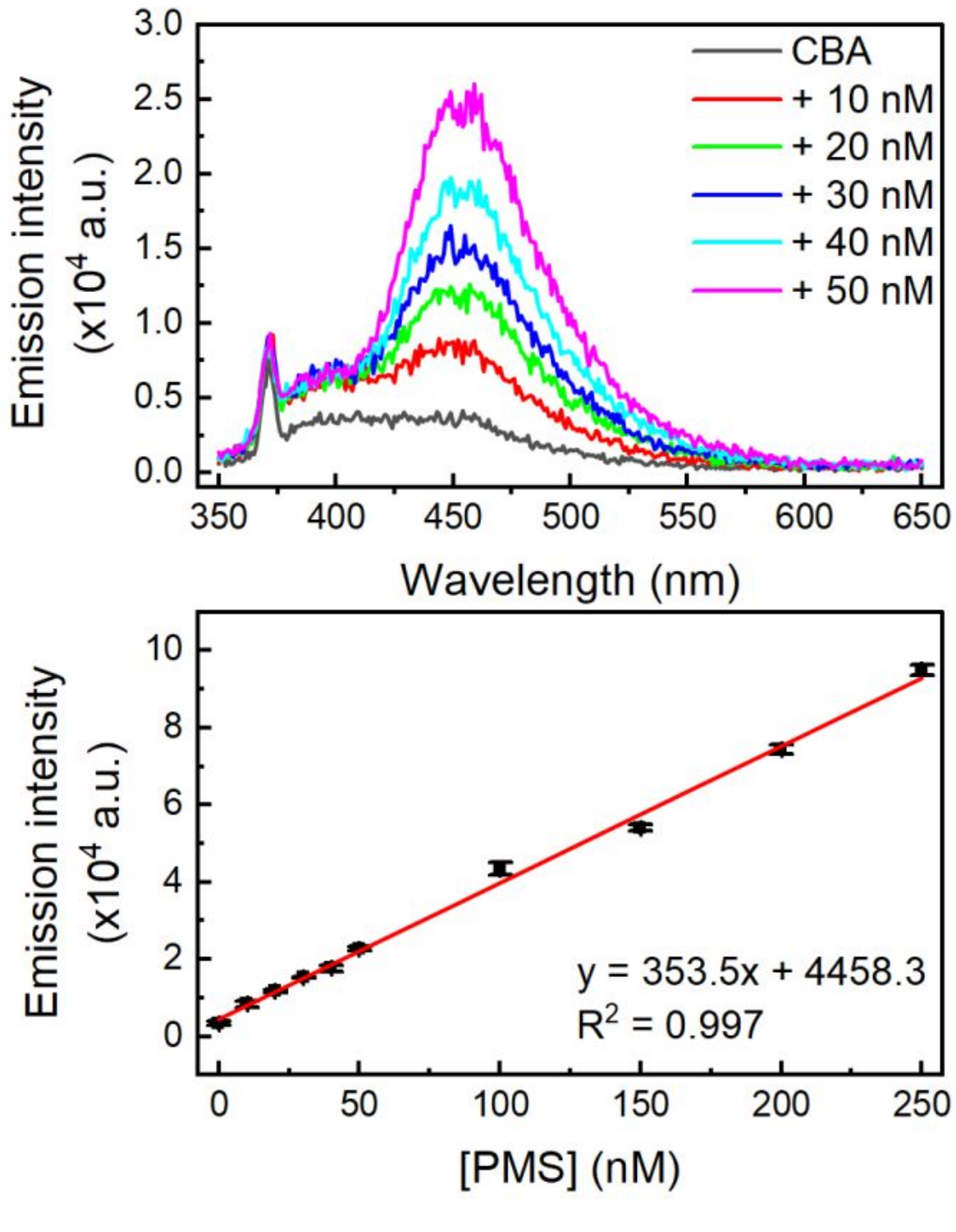

Figure S2. Fluorescence response of CBA (10 μM) after addition of PMS (10 - 250 nM). Experiments were carried out in phosphate buffer (pH 6.8, 0.1 M) with dtpa (10 μM) and MeCN (0.1%). Each spectrum results from three scans. Spectra were recorded after 2 minutes of incubation at room temperature.

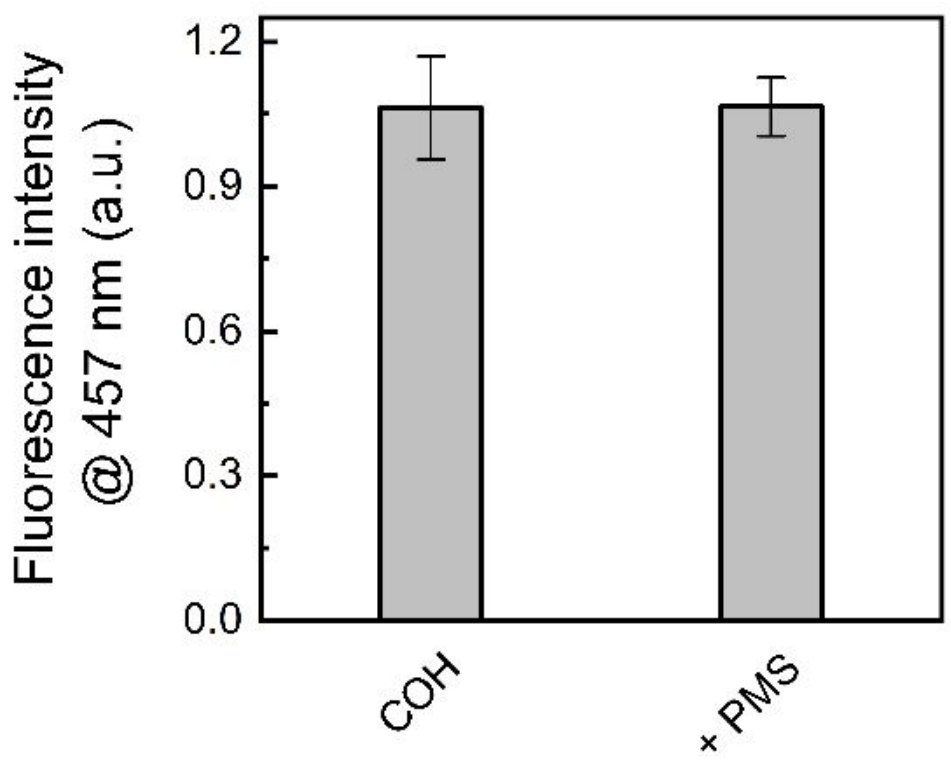

Figure S3. Stability of COH in the presence of PMS. Effect of PMS (2 μM) on the fluorescence signal intensity of COH (11 μM). Experiments were carried out in phosphate buffer (pH 6.8, 0.1 M) with dtpa (10 μM) and MeCN (0.1%). Error bars represent standard deviations from triplicates.

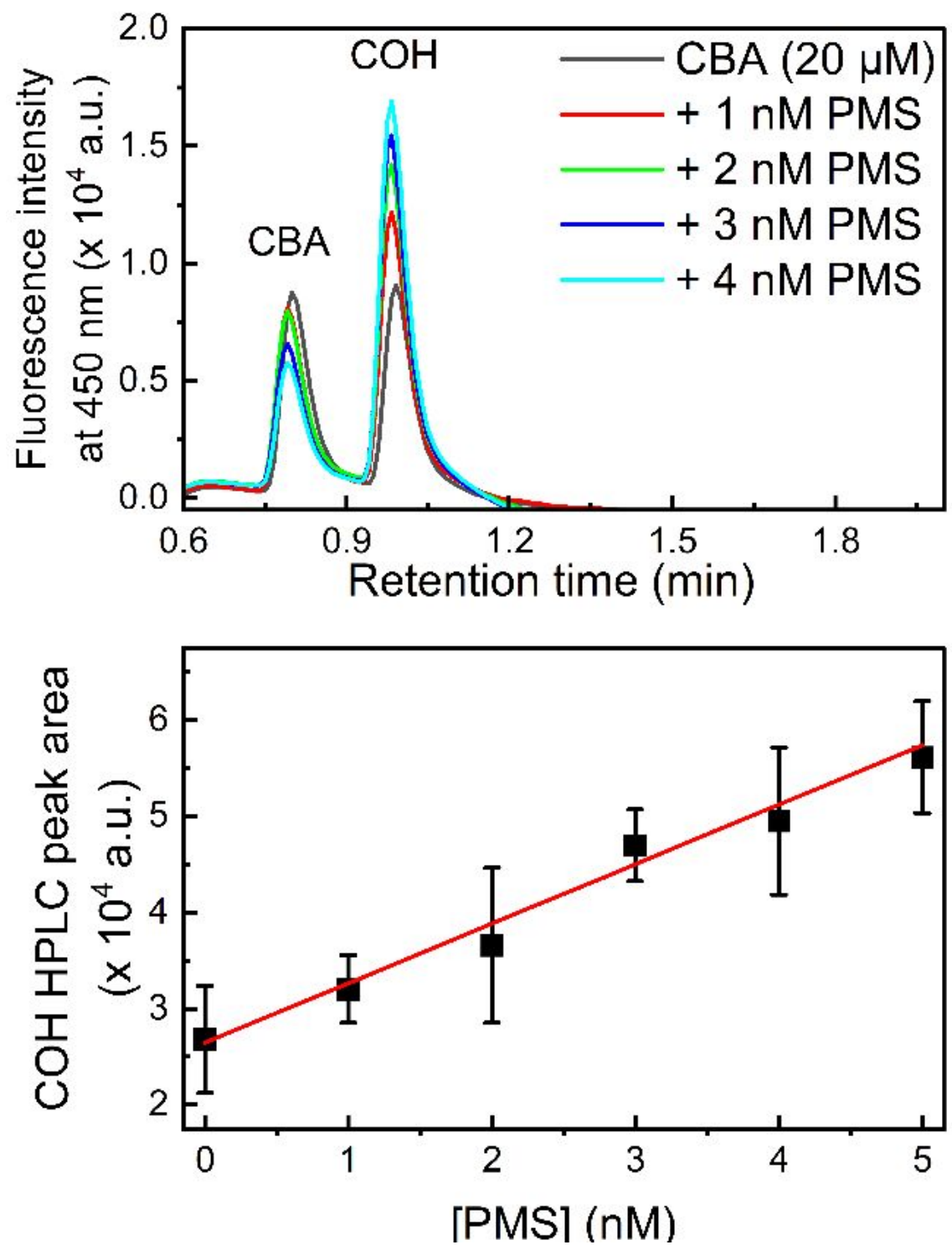

Figure S4. (a) HPLC-FLD chromatograms recorded after adding different concentrations of PMS (1-5 nM) to the CBA probe (20 μM) in the phosphate buffer solution (50 mM, pH 6.8) containing MeCN (0.1%) and dtpa (10 μM). HPLC-FLD traces were recorded after 2 minutes of incubation at 25 °C, with excitation and emission set at 330 nm and 450 nm, respectively; (b) The dependence of the peak area of the COH signal on the PMS concentration (1 - 5 nM). Error bars indicate standard deviations of triplicates.

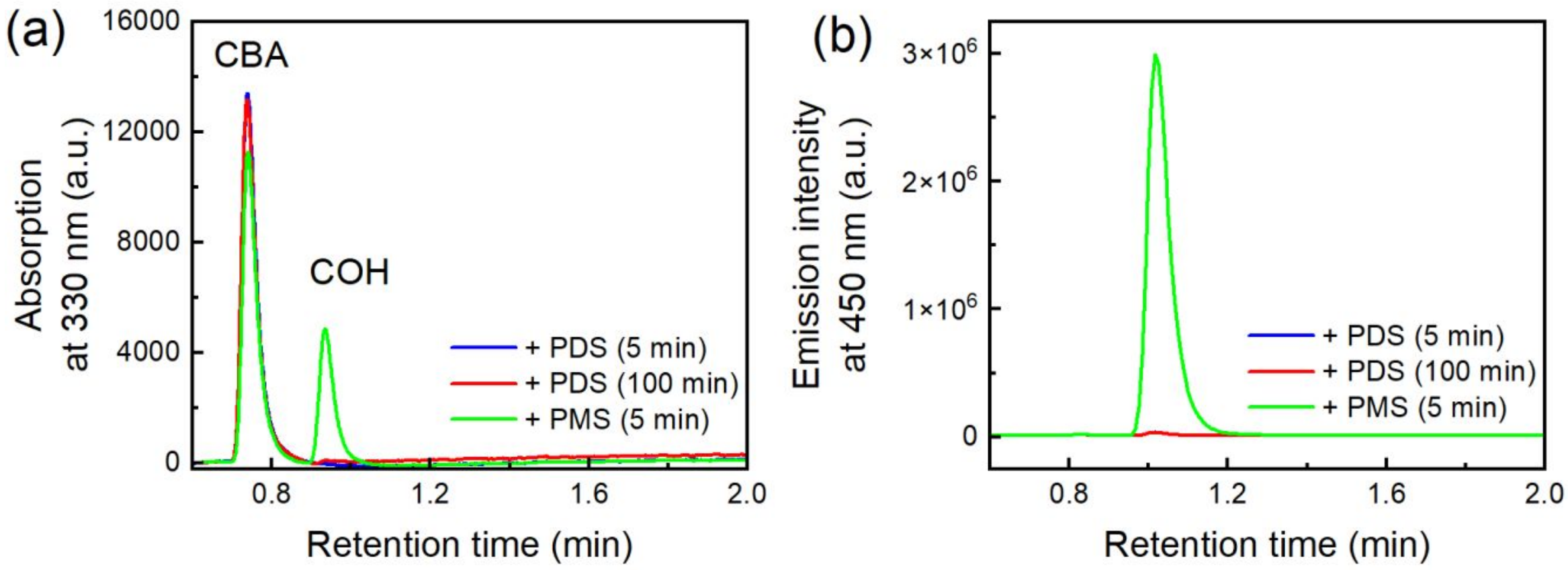

Figure S5. Comparison of CBA reactivity toward peroxymonosulfate (PMS) and peroxydisulfate (PDS). Chromatograms of reaction mixtures after 5 and 100 minute incubation in a phosphate buffer (pH 6.8, 10 mM) with dtpa (10 μM) and MeCN (0.2%) containing CBA (20 μM) and PMS (2 μM) or PDS (2 μM) were recorded using (a) absorption and (b) fluorescence detectors.

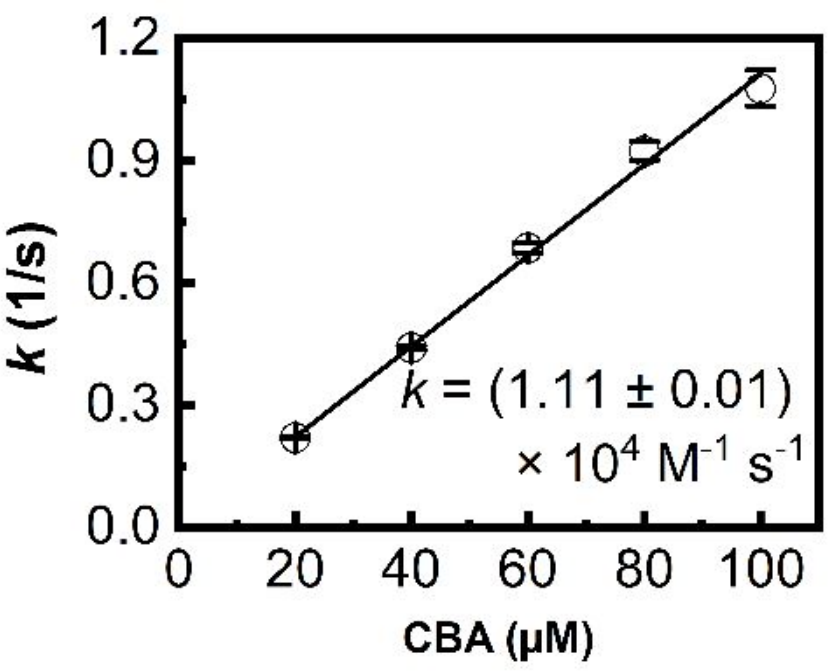

Figure S6. Kinetics of the reaction between CBA and PMS. The dependence of pseudo-first-order rate constants of the reaction of CBA with PMS on the CBA concentration. The pseudo-first-order rate constants were obtained by fitting the build-up of the signal at 470 nm corresponding to COH formation in the reaction between CBA (20-100 μM) and HSO_5^- (5 μM) in an aqueous solution containing a phosphate buffer (50 mM, pH 7.4), CH_3CN (10%), and dtpa (200 μM). Error bars indicate standard deviations of triplicates.

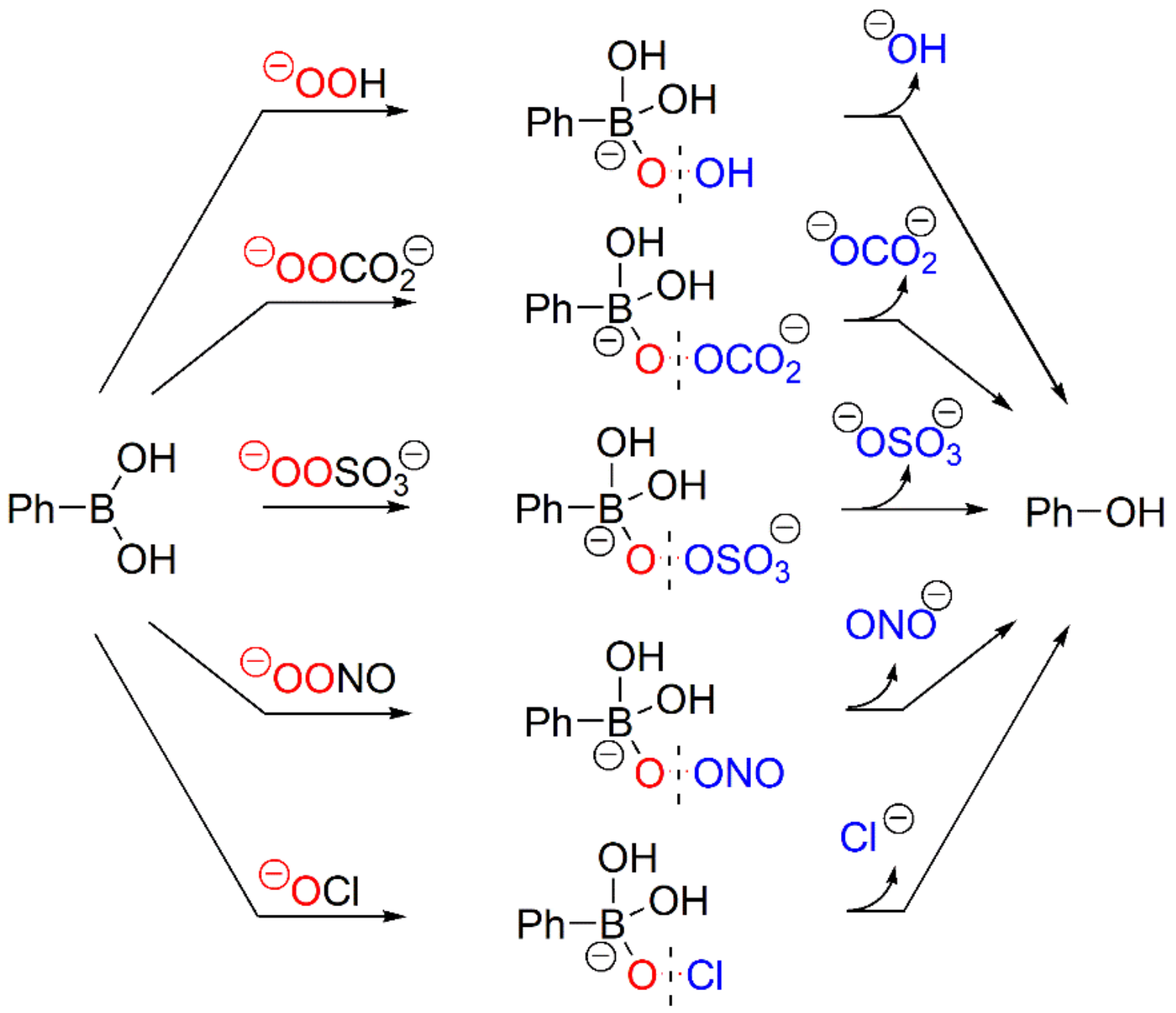

Scheme S1. General mechanism of phenylboronic acid oxidation induced by anionic peroxides and the anion of hypochlorous acid, with the leaving groups marked in blue.

Table S2. Comparison of the properties of PMS with other peroxy-oxidants.

Oxidant	pK_a [10]	at pH = 7.4 [a] (%)	A [b]	^2k [10] $ArB(OH)_2$ + oxidant ($M^{-1}s^{-1}$)	$\Delta G_{hyd}(A)$ (kcal mol $^{-1}$)
H_2O_2	11.7	0.005	HO^-	~ 1×10^0	-104.5 [11]<br>-107.0 [12]
O_2COOH	10.6 [13]	0.06	CO_3^{2-}	~ $1 - 2 \times 10^2$ [14]	-314.0 [15]
$^-O_3SOOH$	9.4<br>9.13 [16]	1<br>1.8	SO_4^{2-}	~ 1×10^4	-266.0 [17]
HOCl	7.5	46	Cl^-	~ 1×10^4	-73.9 [18]<br>-73.0 [12]
ONOOH	6.8	80	NO_2^-	~ 1×10^6	-69.3 [19]

[a] calculated fractions of the boronate-reactive anionic forms; [b] leaving group (see scheme S1);

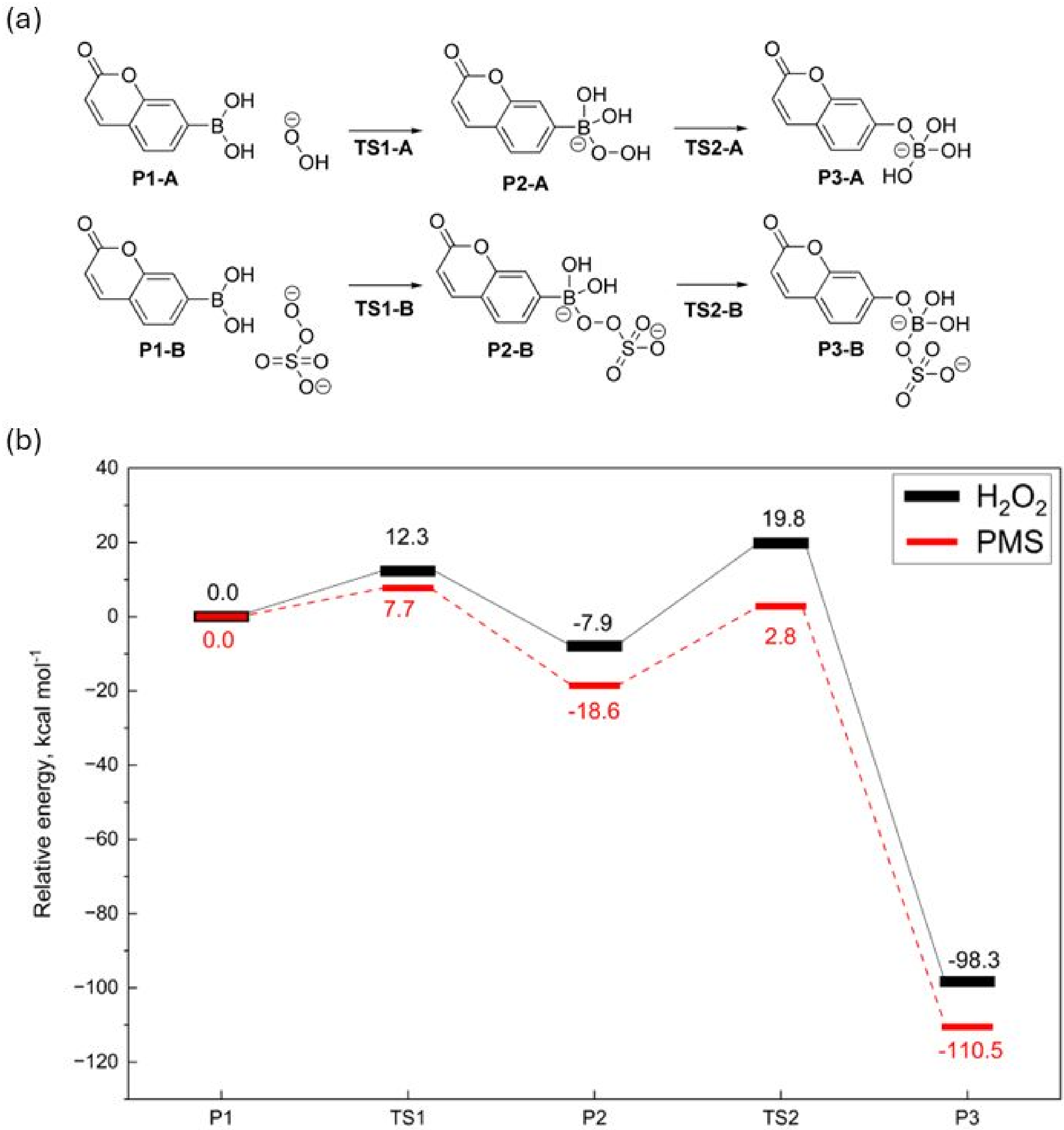

Figure S7. Results of DFT calculations. (a) The nucleophilic addition of the HOO^- and SO_5^{2-} to the boron atom with the formation of P2-A and P2-B complexes followed by their rearrangements to P3-A and P3-B anions. Water molecules were omitted for clarity; (b) Energy diagrams for the first two steps of the oxidation of CBA by H_2O_2 or PMS.

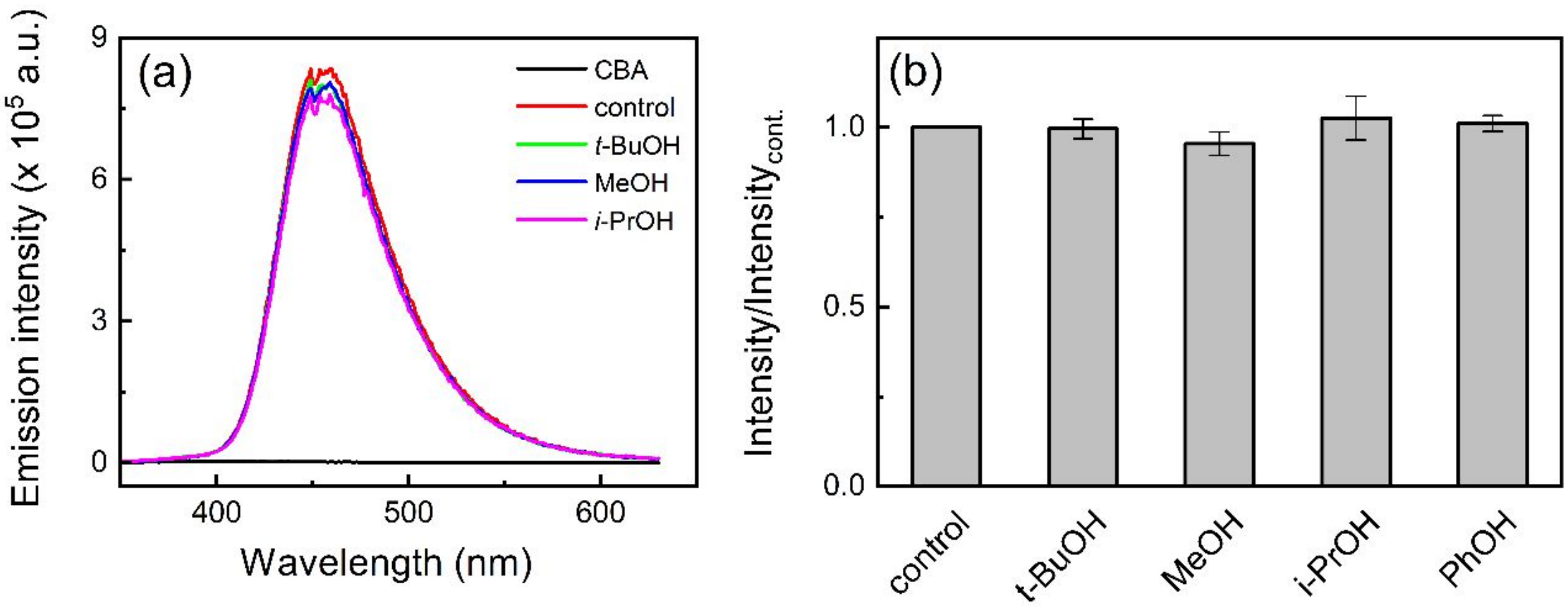

Figure S8. Effect of additives on the fluorescent response of CBA toward PMS. (a) Representative fluorescence spectra of the CBA probe (100 μM) alone and after its oxidation by PMS (80 μM) under different conditions, in a phosphate buffer (pH 6.8, control) and with the addition of alcohols (phosphate buffer, pH 6.8: alcohol (5%)). Spectra were recorded after a 10-fold dilution of the reaction mixture; (b) effect of alcohols (5%) and phenol (1.25 μM) on the oxidation of CBA by PMS.

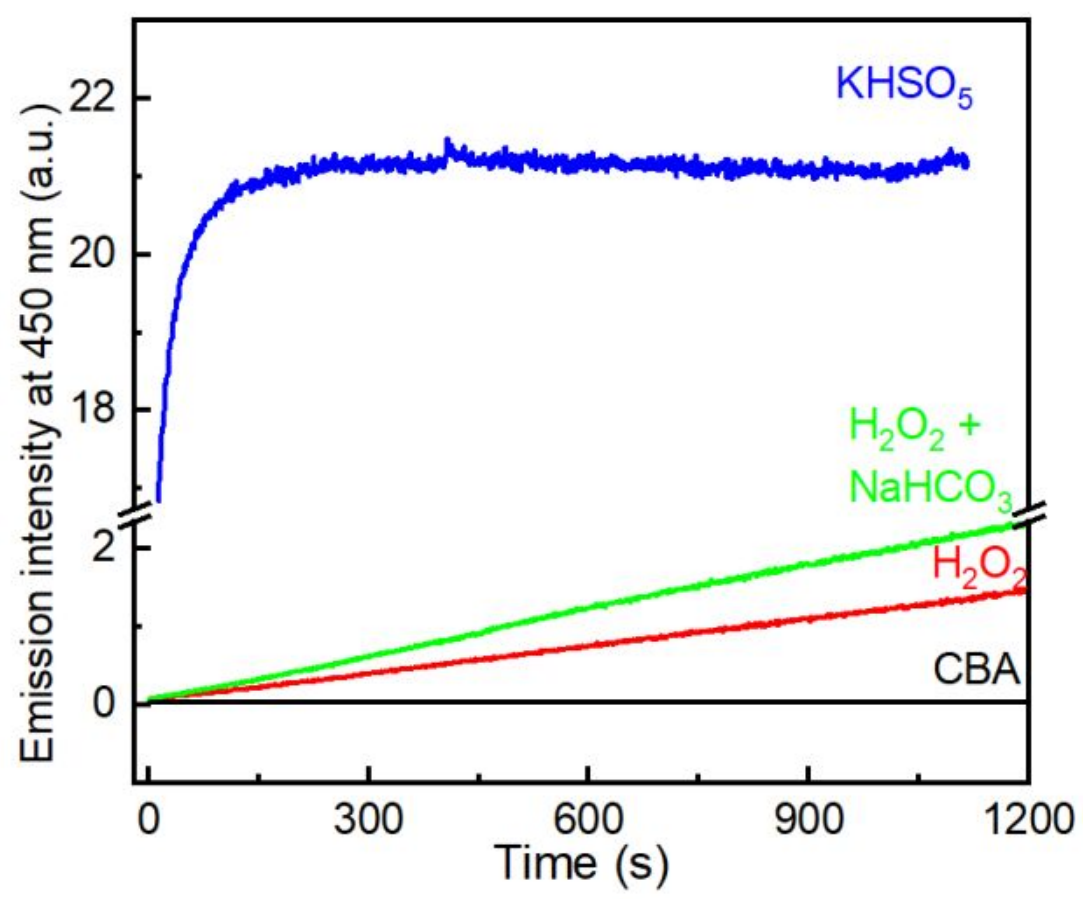

Figure S9. Kinetic profiles of COH formation during the oxidation of CBA (10 μM) by PMS (75 μM), H_2O_2 (200 μM), and H_2O_2 (200 μM) in the presence of $NaHCO_3$ (200 μM).

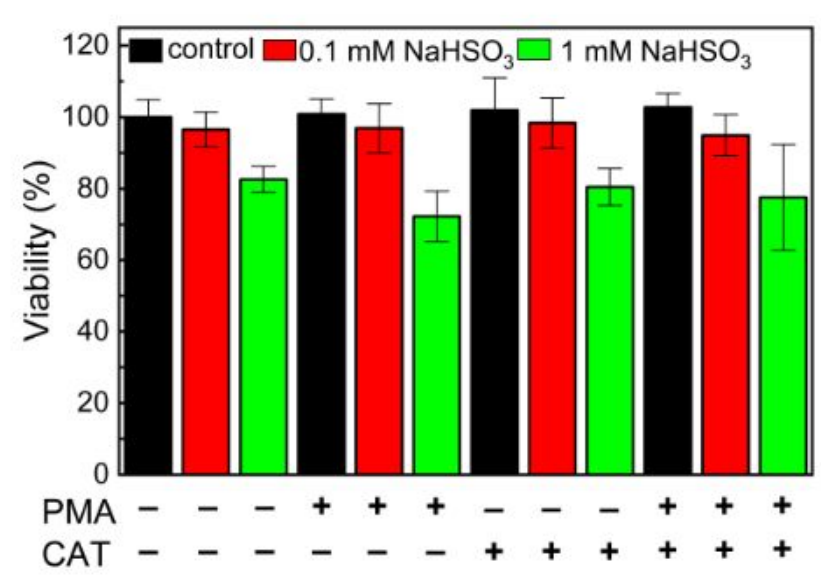

Figure S10. Effect of $NaHSO_3$, in the presence or absence of PMA and CAT, on the metabolic activity of RAW 264.7 cells. Cell viability was assessed using the CellTiter-Glo luminescent assay [9] after 2 h incubation with the tested compounds and the CBA probe.

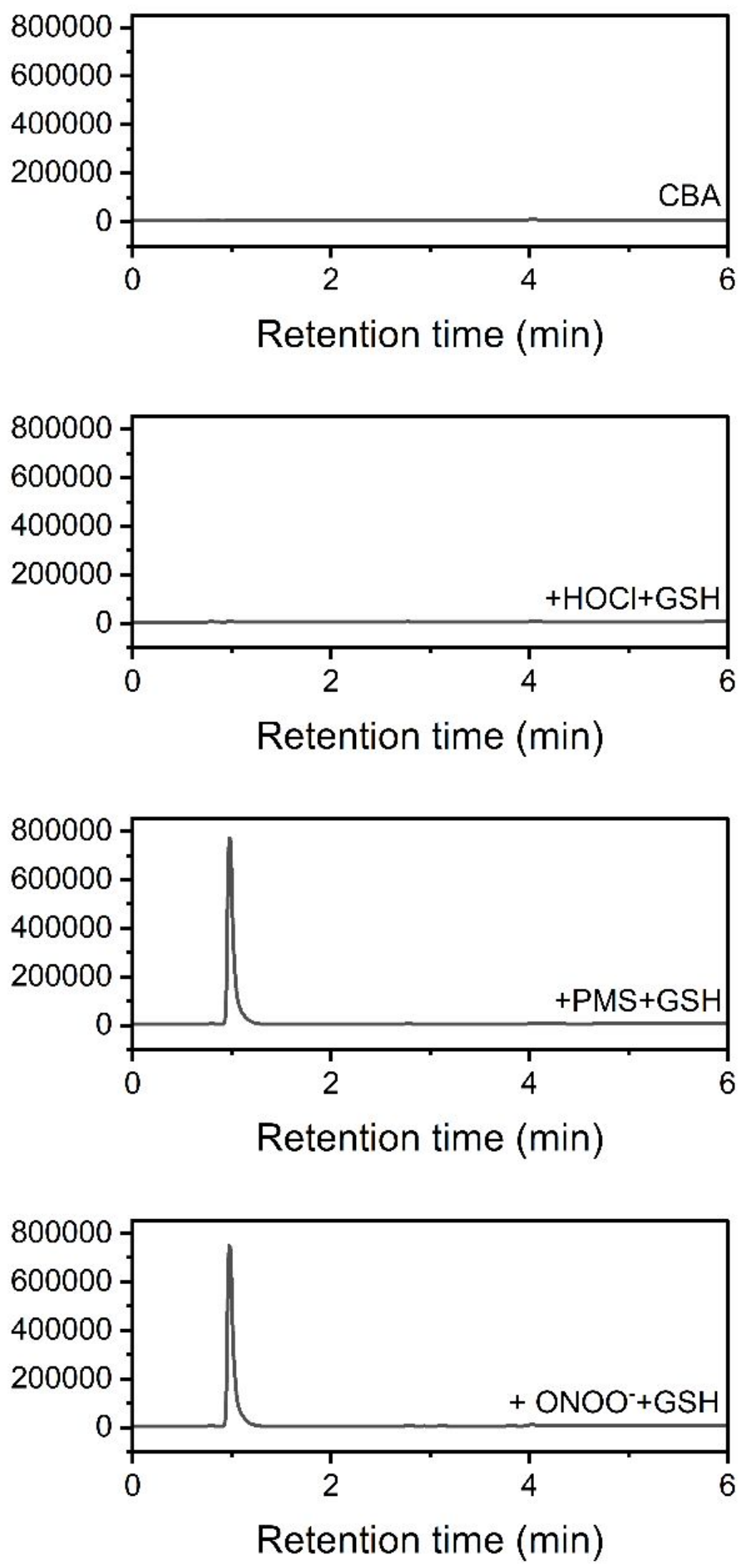

Figure S11. Effect of GSH on the oxidation of the CBA probe by HOCl, PMS, and peroxynitrite. HPLC-FLD chromatograms recorded for the solution of CBA (10 μM) with glutathione (GSH, 100 μM) and after mixing with HOCl (1 μM), PMS (1 μM), or peroxynitrite (1 μM). HPLC-FLD traces were recorded after 2 minutes of incubation at 25 °C in the phosphate buffer solution (50 mM, pH 6.8) containing MeCN (0.1%), dtpa (10 μM), and with excitation and emission set at 330 nm and 450 nm, respectively.

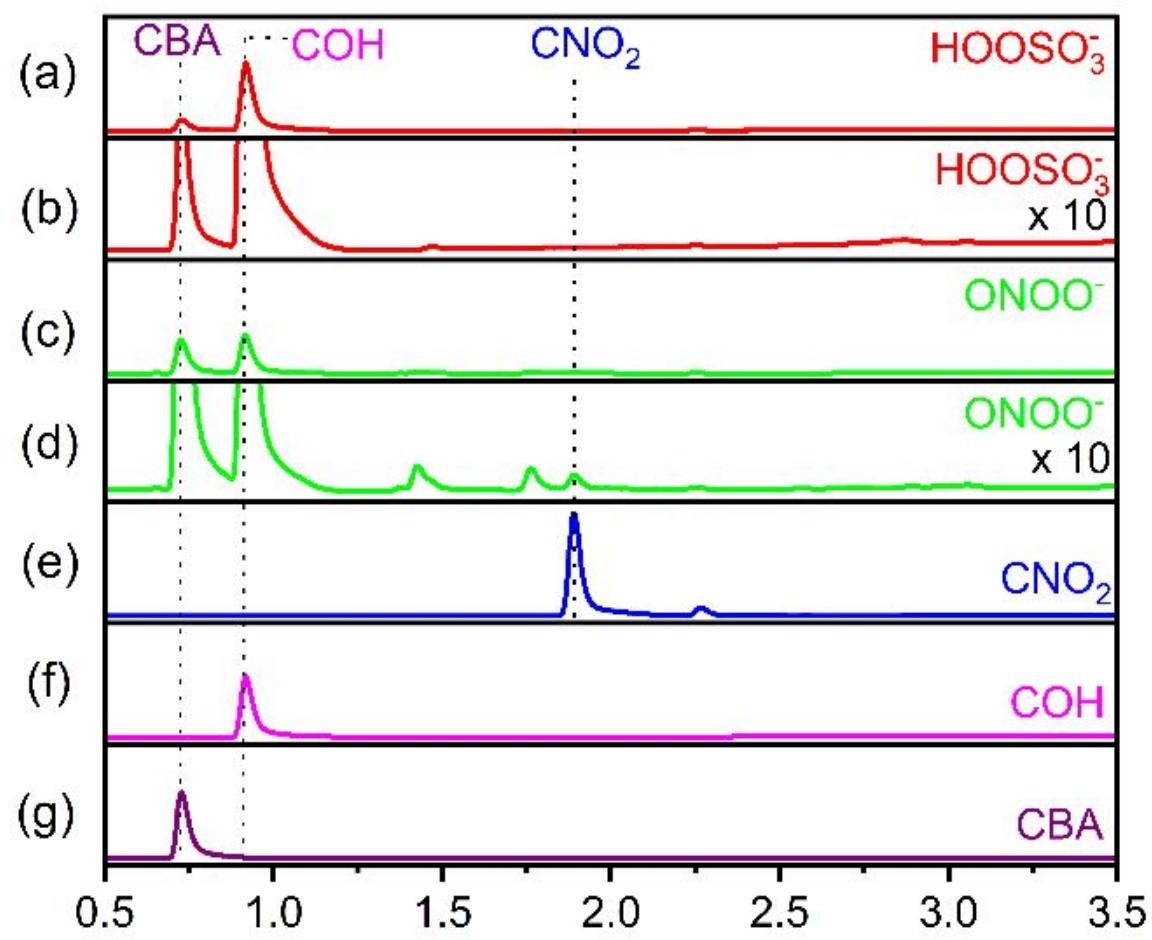

Figure S12. Comparison of the products formed during the reaction of the CBA probe with PMS and peroxynitrite. HPLC-PDA traces recorded at 300 nm (a) CBA (100 μM) with the addition of PMS (80 μM) in phosphate buffer (50 mM, pH 6.8); (b) the same as in (a), but 10 times enlarged; (c) CBA (100 μM) with the addition of peroxynitrite (40 μM) in phosphate buffer (50 mM, pH 6.8); (d) the same as in (c), but 10 times enlarged; (e) 7-nitrocoumarin, CNO_2 (100 μM) (f) 7-hydroxycoumarin, COH (100 μM) (g) coumarin-7-boronic acid, CBA (100 μM) in a phosphate buffer (50 mM, pH 6.8).

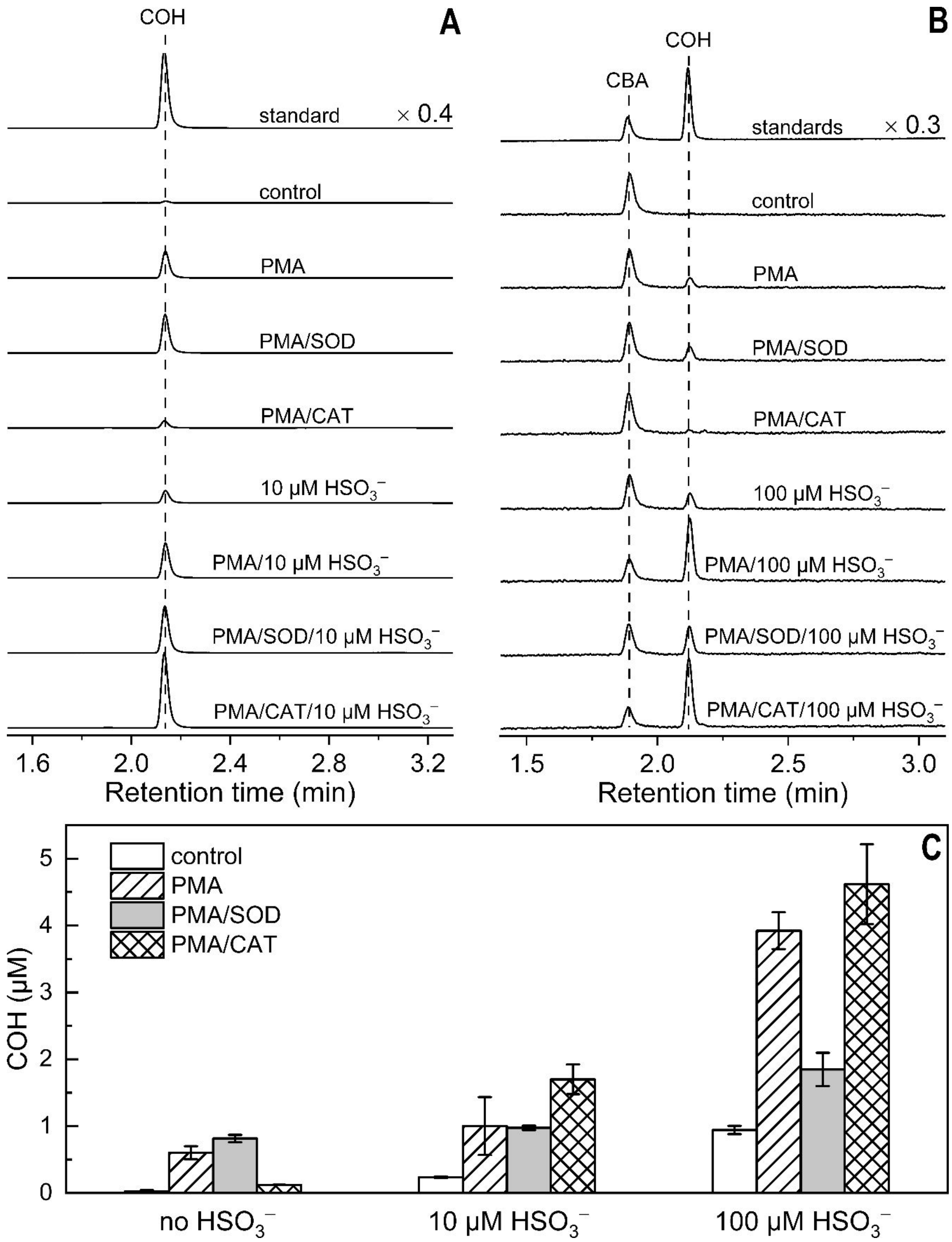

Figure S13. UPLC detection of PMS in RAW 264.7 cells. (A) UPLC traces obtained from the culture media of RAW 264.7 cells incubated in the presence or absence of CBA (20 μM), PMA (1 μM), SOD (0.1 mg/mL), catalase (1 kU/mL) and bisulfite (10 μM). The concentration of the COH standard was 1 μM. The UPLC traces were recorded using fluorescence detector (λ_{ex} = 332 nm and λ_{em} = 450 nm). (B) Same as in (A), except that the UPLC traces were recorded using a PDA detector at 320 nm, the concentration of bisulfite was 100 μM, and both CBA (5 μM) and COH (5 μM), standards were used. (C) Concentrations of COH detected in the culture media from the RAW 264.7 incubations.

DFT calculations

Density functional theory (DFT) calculations were performed to provide molecular-level insight into the oxidation mechanisms investigated in this study. Initial transition-state (TS) geometries reported previously by Gatin-Fraudet et al. [20], consisting of phenylboronic acid, the hydroperoxide anion (HOO^-), and ten explicitly included water molecules, were used as starting geometries for constructing the corresponding CBA models reacting with experimentally studied oxidizing species, namely hydroperoxide anion and peroxymonosulfate dianion (SO_5^{2-}).

For newly optimized transition state structures (TSs), intrinsic reaction coordinate (IRC) calculations were carried out to identify the neighboring energy minima on the potential energy surface (PES), corresponding to the closest reactant and product complexes. Their geometries were then fully reoptimized.

All calculations were carried out using tight convergence criteria. Harmonic vibrational frequency calculations were performed for all optimized structures to verify their nature as transition states (single imaginary frequency corresponding to the reaction coordinate) or minima (no imaginary frequencies), and to obtain thermochemical corrections for the calculation of Gibbs free energies. Relative Gibbs free energies (ΔG) reported in Table S3 are referenced to the first stationary point (P1) along each reaction pathway. For comparison, calculations for the oxidation of phenylboronic acid by both oxidants are also included, reproducing previously reported results for hydroperoxide anion.[20]

All calculations were performed using the Gaussian 16 software package, Revision C.02. [21]

Table S3. Relative Gibbs free energies (ΔG, $kcal \cdot mol^{-1}$) of the stationary points located along the reaction pathways investigated. Energies are referenced to the reactant complex (P1) of the corresponding pathway.

	**CBA**		**Phenylboronic acid**	
	**HOO^-**	**SO_5^{2-}**	**HOO^-**	**SO_5^{2-}**
**P1**	0.0	0.0	0.0	0.0
**TS1**	12.3	7.7	13.2	8.1
**P2**	-7.9	-18.6	-7.2	-17.7
**TS2**	19.8	2.8	18.8	1.9
**P3**	-98.3	-110.5	-97.3	-108.9

References

[1] P. E. A. Boudeville, Simultaneous determination of hydrogen peroxide, peroxymonosulfuric acid, and peroxydisulfuric acid by thermometric titrimetry, Anal. Chem. 1983, 55, 612-615. DOI:10.1021/ac00255a007

[2] T. Zhang, H. Zhu, J.-P. Croué, Production of Sulfate Radical from Peroxymonosulfate Induced by a Magnetically Separable $CuFe_2O_4$ Spinel in Water: Efficiency, Stability, and Mechanism, Environ. Sci. Technol. 2013, 47, 2784-2791. DOI:10.1021/es304721g

[3] V.K. Zinchuk, E.N. Stadnichuk, Chemiluminescence Determination of Monopersulfuric Acid, J. Anal. Chem. 2001,56, 918-919. DOI:10.1023/A:1012353226910

[4] S. Wacławek, K. Grübel, M. Černik, Simple spectrophotometric determination of monopersulfate, Spectrochim. Acta A 2015, 149, 928-933. DOI:10.1016/j.saa.2015.05.029

[5] S. Gokulakrishnan, A. Mohammed, H. Prakash, Determination of persulphates using N,N-diethyl-p-phenylenediamine as colorimetric reagent: Oxidative coloration and degradation of the reagent without bactericidal effect in water, Chem. Eng. J. 2016, 286, 223-231. DOI:10.1016/j.cej.2015.10.058

[6] J. Zou, J. Ma, X. Zhang, P. Xie, Rapid spectrophotometric determination of peroxymonosulfate in water with cobalt-mediated oxidation decolorization of methyl orange, Chem. Eng. J. 2014, 253, 34-39. DOI:10.1016/j.cej.2014.05.042

[7] J. Q. Zhang, J. Ma, J. Zou, H. Z. Chi, Y. Song, Spectrophotometric determination of peroxymonosulfate anions via oxidative decolorization of dyes induced by cobalt, Anal. Methods, 2016, 8, 973-978. DOI:10.1039/C5AY02886H

[8] G.-X. Huang, J.-Y. Si, C. Qian, W.-K. Wang, S.-C. Mei, C.-Y. Wang, H.-Q. Yu, Ultrasensitive Fluorescence Detection of Peroxymonosulfate Based on a Sulfate Radical-Mediated Aromatic Hydroxylation. Anal. Chem. 2018, 90, 14439-14446. https://doi.org/10.1021/acs.analchem.8b04047

[9] Hannah, R.; Beck, M.; Moravec, R.; Riss, T. CellTiter-Glo™ Luminescent Cell Viability Assay: A Sensitive and Rapid Method for Determining Cell Viability. *Promega Cell Notes* **2001**, *2*.

[10] Sikora, A.; Zielonka, J.; Dębowska, K.; Michalski, R.; Smulik-Izydorczyk, R.; Pięta, J.; Podsiadły, R.; Artelska, A.; Pierzchała, K.; Kalyanaraman, B. Boronate-Based Probes for Biological Oxidants: A Novel Class of Molecular Tools for Redox Biology. Front. Chem. 2020, 8, 580899. https://doi.org/10.3389/fchem.2020.580899.

[11] Zhan, C.-G.; Dixon, D. A. First-Principles Determination of the Absolute Hydration Free Energy of the Hydroxide Ion. J. Phys. Chem. A 2002, 106 (42), 9737-9744. https://doi.org/10.1021/jp014533l.

[12] Kelly, C. P.; Cramer, C. J.; Truhlar, D. G. SM6: A Density Functional Theory Continuum Solvation Model for Calculating Aqueous Solvation Free Energies of Neutrals, Ions, and Solute−Water Clusters. J. Chem. Theory Comput. 2005, 1 (6), 1133-1152. https://doi.org/10.1021/ct050164b.

[13] Balagam, B.; Richardson, D. E. The Mechanism of Carbon Dioxide Catalysis in the Hydrogen Peroxide N-Oxidation of Amines. Inorg. Chem. 2008, 47 (3), 1173-1178. https://doi.org/10.1021/ic701402h.

[14] Linares, E.; Severino, D.; Truzzi, D. R.; Rios, N.; Radi, R.; Augusto, O. Production of Peroxymonocarbonate by Steady-State Micromolar H_2O_2 and Activated Macrophages in the Presence of CO_2/HCO_3^- Evidenced by Boronate Probes. Chem. Res. Toxicol. 2024, 37 (7), 1129-1138. https://doi.org/10.1021/acs.chemrestox.4c00059.

[15] Raiteri, P.; Schuitemaker, A.; Gale, J. D. Ion Pairing and Multiple Ion Binding in Calcium Carbonate Solutions Based on a Polarizable AMOEBA Force Field and Ab Initio Molecular Dynamics. J. Phys. Chem. B 2020, 124 (17), 3568-3582. https://doi.org/10.1021/acs.jpcb.0c01582

[16] Amels, P.; Elias, H.; Wannowius, K.-J. Kinetics and Mechanism of the Oxidation of Dimethyl Sulfideby Hydroperoxides in Aqueous Medium Study on the Potentialcontribution of Liquid-Phase Oxidation of Dimethyl Sulfide in Theatmosphere. J. Chem. Soc., Faraday Trans. 1997, 93 (15), 2537-2544. https://doi.org/10.1039/A700722A.

[17] Cannon, W. R.; Pettitt, B. M.; McCammon, J. A. Sulfate Anion in Water: Model Structural, Thermodynamic, and Dynamic Properties. J. Phys. Chem. 1994, 98 (24), 6225-6230. https://doi.org/10.1021/j100075a027.

[18] Sündermann, A.; Reif, M. M.; Hofbauer, S.; Obinger, C.; Oostenbrink, C. Investigation of Ion Binding in Chlorite Dismutases by Means of Molecular Dynamics Simulations. Biochemistry 2014, 53 (29), 4869-4879. https://doi.org/10.1021/bi500467h.

[19] Camaioni, D. M.; Dupuis, M.; Bentley, J. Theoretical Characterization of Oxoanion, XO_m^{n-}, Solvation. J. Phys. Chem. A 2003, 107 (30), 5778-5788. https://doi.org/10.1021/jp0343537.

[20] Gatin-Fraudet, B., Ottenwelter, R., Le Saux, T., Norsikian, S., Pucher, M., Lombès, T., Baron, A., Durand, P., Doisneau, G., Bourdreux, Y., Iorga, B.I., Erard, M., Jullien, L., Guianvarc'h, D., Urban, D., Vauzeilles, B., Evaluation of borinic acids as new, fast hydrogen peroxide–responsive triggers, Proc. Natl. Acad. Sci. U.S.A. 2021, 118 (50) e2107503118, https://doi.org/10.1073/pnas.2107503118.

[21] Gaussian 16, Revision C.02, Frisch, M. J.; Trucks, G. W.; Schlegel, H. B.; Scuseria, G. E.; Robb, M. A.; Cheeseman, J. R.; Scalmani, G.; Barone, V.; Petersson, G. A.; Nakatsuji, H.; Li, X.; Caricato, M.; Marenich, A. V.; Bloino, J.; Janesko, B. G.; Gomperts, R.; Mennucci, B.; Hratchian, H. P.; Ortiz, J. V.; Izmaylov, A. F.; Sonnenberg, J. L.; Williams-Young, D.; Ding, F.; Lipparini, F.; Egidi, F.; Goings, J.; Peng, B.; Petrone, A.; Henderson, T.; Ranasinghe, D.; Zakrzewski, V. G.; Gao, J.; Rega, N.; Zheng, G.; Liang, W.; Hada, M.; Ehara, M.; Toyota, K.; Fukuda, R.; Hasegawa, J.; Ishida, M.; Nakajima, T.; Honda, Y.; Kitao, O.; Nakai, H.; Vreven, T.; Throssell, K.; Montgomery, J. A., Jr.; Peralta, J. E.; Ogliaro, F.; Bearpark, M. J.; Heyd, J. J.; Brothers, E. N.; Kudin, K. N.; Staroverov, V. N.; Keith, T. A.; Kobayashi, R.; Normand, J.; Raghavachari, K.; Rendell, A. P.; Burant, J. C.; Iyengar, S. S.; Tomasi, J.; Cossi, M.; Millam, J. M.; Klene, M.; Adamo, C.; Cammi, R.; Ochterski, J. W.; Martin, R. L.; Morokuma, K.; Farkas, O.; Foresman, J. B.; Fox, D. J. Gaussian, Inc., Wallingford CT, 2016.
